# Prediction of dengue outbreak in Selangor Malaysia using machine learning techniques

**DOI:** 10.1038/s41598-020-79193-2

**Published:** 2021-01-13

**Authors:** Nurul Azam Mohd Salim, Yap Bee Wah, Caitlynn Reeves, Madison Smith, Wan Fairos Wan Yaacob, Rose Nani Mudin, Rahmat Dapari, Nik Nur Fatin Fatihah Sapri, Ubydul Haque

**Affiliations:** 1 Advanced Analytics Engineering Centre, Faculty of Computer and Mathematical Sciences, Universiti Teknologi MARA, 40450 Shah Alam, Selangor Malaysia; 2 Faculty of Computer and Mathematical Sciences, Universiti Teknologi MARA Cawangan Kelantan, Kampus Kota Bharu, Lembah Sirrh, 15050 Kota Bharu, Kelantan Malaysia; 3 Department of Biostatistics and Epidemiology, University of North Texas Health Science Center, Fort Worth, TX 76107 USA; 4 Vector Borne Disease Sector, Disease Control Division, Ministry of Health Malaysia, Level 4, Block E10, Complex E, Federal Government Administration Complex, 62590 Putrajaya, Malaysia

**Keywords:** Ecology, Risk factors

## Abstract

Dengue fever is a mosquito-borne disease that affects nearly 3.9 billion people globally. Dengue remains endemic in Malaysia since its outbreak in the 1980’s, with its highest concentration of cases in the state of Selangor. Predictors of dengue fever outbreaks could provide timely information for health officials to implement preventative actions. In this study, five districts in Selangor, Malaysia, that demonstrated the highest incidence of dengue fever from 2013 to 2017 were evaluated for the best machine learning model to predict Dengue outbreaks. Climate variables such as temperature, wind speed, humidity and rainfall were used in each model. Based on results, the SVM (linear kernel) exhibited the best prediction performance (Accuracy = 70%, Sensitivity = 14%, Specificity = 95%, Precision = 56%). However, the sensitivity for SVM (linear) for the testing sample increased up to 63.54% compared to 14.4% for imbalanced data (original data). The week-of-the-year was the most important predictor in the SVM model. This study exemplifies that machine learning has respectable potential for the prediction of dengue outbreaks. Future research should consider boosting, or using, nature inspired algorithms to develop a dengue prediction model.

## Introduction

Dengue fever is a re-emerging, mosquito-borne, viral disease with over 3.9 billion individuals at risk of infection worldwide^1^. The disease is endemic in 128 countries throughout South Asia, South-East Asia, Africa, the Americas, the Western Pacific and Eastern Mediterranean regions^2–4^.

Malaysia, a Southeast Asian country, has experienced cases of dengue since 1902. The disease became a public health risk in the 1970’s, with its first major outbreak in 1973^5,6^. The incidence of dengue fever has increased from 32 cases per 100,000 individuals in the year 2000 to 361 cases per 100,000 population in 2014^7^. Most individuals afflicted with dengue are between the ages of 15 and 49, and 80% of cases are within urban communities^7^. Selangor is a densely populated and urban state in Malaysia, containing 5.79 million of the country’s 31.53 million inhabitants, and contributing to 90% of national dengue cases^7^.

Based on the systematic review by Louis et al., risk mapping studies have mostly been descriptive, lacking validation and predictive value. Hence, there is a need for additional tools, such as studying climate and mobility data in dengue prediction. Climatic data and weather data were often used in the generation of predictive risk maps and modeling dengue incidence^8^.

Researchers have found that the transmission of dengue fever is largely affected by inter-annual and seasonal climate variability^9–12^. For example, the temperature factor has been found to be a significant climate variable in contributing to the incidence of dengue fever. An environmental-controlled experiment discovered temperature provides the optimal environment for survival of adult mosquitoes as well as for larva, pupal and egg (in aquatic phase)^13^.

Malaysia continues to lack thorough comparisons of different predictive models and an identification of an optimal model. Furthermore, this study seeks to identify spatiotemporal dengue hotspot areas in Selangor, Malaysia, determine the association of climate variables with dengue fever outbreak, and evaluate machine learning models for predicting dengue fever outbreaks.

## Material and methods

### Source of data

The data contains 5 years (2013 to 2017) of weekly case numbers for five districts in Selangor, Malaysia: Gombak, Hulu Selangor, Hulu Langat, Klang, and Petaling. After verification and validation by the district health staff, all notified cases with clinical symptoms of dengue, and confirmed dengue laboratory results (either NS1 positive, IgM positive and IgG positive results through rapid test kit, PCR confirmed, ELISA serology confirmed, or virus isolation), are registered in eNotifikasi, a real-time surveillance system. Cases from private clinics, public clinics, and hospitals are also reported to the Ministry of Health through eNotifikasi. Once the case registered is in the eNotifikasi system, the dengue case information (e.g., name, identification card number, date of birth, address, age, date of onset, and date of notification) is transferred into the eDengueV2 system (supplement text, Supplement Fig. 1). The information used in this study is extracted from the eDengueV2 system (More details are in supplement texts and Supplement Tables 1–7).

In Malaysia, annually less than 1% of cases were dengue hemorrhagic syndrome or dengue shock syndrome (Source: Ministry of Health, Malaysia, unpublished report, personal communication Dr. Rose Nani). Based on the new WHO classification of dengue, dengue hemorrhagic syndrome and dengue shock syndrome are classified as “severe” cases of dengue.

Climate data, such as humidity, rainfall, temperature and wind speed, was obtained from the Malaysian Meteorology Department. The description of the variables is shown in Tables 1 and 2 (Supplement). The ‘*AvgRain’, ‘AvgTemp’, ‘Max Temp’, ‘Min Temp’, ‘AvgHumid’,* and *‘AvgWind’* are continuous variables, whereas the ‘*District’,* ‘*Year’* and ‘*Weekofyear*’ are categorical variables. The target variable is a binary variable termed *‘Outbreak’*.

An outbreak is defined as the occurrence of a disease in higher frequency than expected in an area during a specified period. According to the World Health Organization (WHO), the operational definition for an outbreak of dengue fever in Malaysia is the reporting of more than two standard deviations of the 4-week-case-average above the moving three 4-week-case-average of dengue cases^14^.

### Data cleaning

Data cleaning was carried out in Microsoft Excel. Missing values were detected in the data during data cleaning, with 6% missing in Temperature, 7% missing in Humidity, 2% missing in Rainfall, and 4% missing in Wind Speed. Missing values within the raw climate dataset were recorded differently by each station and were corrected using data imputation. The Climatological Mean of the Day (CMD) method, viewing the available data at hand, uses an average of the previous daily value on the same day. Calculation of the estimated value (V_est_) is as follow:

$${\mathrm{V}}_{\mathrm{est}}=\frac{{\sum }_{j=1}^{T}{V}_{ij}}{T}$$

where, V_i_ is the value of the variable for the ith day of year j and T is the number of available data for that year^15^. For example, if rainfall data for Day 5 is missing, the average of all available rainfall data for day 5 of that specific year is taken.

### Creating the dengue outbreak variable

The target variable (dengue outbreak) indicates whether there was a dengue outbreak in a particular week-of-the-year in each district. In establishing this target variable, the WHO operational definition was adopted for dengue fever outbreak in Malaysia. The WHO defines a dengue outbreak as a period of time in which a reported case of a week is more than the sum of the moving average of three 4-week dengue cases plus the value of two standard deviations above the number of dengue cases for the cases four weeks prior^16^. The dengue outbreak variable (1 = Dengue Outbreak, 0 = No dengue outbreak) was created based on the original variable of reported number of dengue cases. Table 3 illustrates (supplement) the steps used to calculate the target variable for the seventh week (Supplement). First, the average number of dengue cases for four weeks prior is calculated. Second, the value for the two-standard deviations above the dengue case number for the four weeks prior is calculated. Third, the moving average of three, 4-week dengue cases is calculated. Fourth, the sum of moving average of the three, 4-week dengue cases plus the two standard deviations of dengue cases for the cases four weeks prior is calculated (Step 3–Step 2). Finally, if the weekly cases are more than the generated value in step 4, then an outbreak has likely occurred. In the seventh week, there are 69 cases of dengue fever and the value generated in step 4 is 80.1. Because the number of cases is less than the calculated number necessary for an outbreak, there appears to be no outbreak of dengue fever in the seventh week. Data cleaning generated 1300 records for the binary “dengue outbreak” variable, and the climate variables for five districts. There are 372 cases for Dengue Outbreak = Yes and 928 cases for Dengue Outbreak = No.

### Model building

Predictive modeling was conducted using IBM SPSS Modeler 18. The cleaned dataset, with 1300 records, was imported into the source node, which was subsequently connected to the data partition node. The data (n = 1300) was partitioned into samples of 70% training and 30% testing. In developing predictive model, this is the standard procedure. The predictive modelling was performed using several data mining models, namely Decision Trees (CART), Artificial Neural Network (MLP), SVM (LINEAR, POLYNOMIAL, RBF), and Bayes Network (TAN). The models were evaluated in the analysis node.

Effectiveness refers to the ability of the classifier to predict the dengue outbreak. A classifier is effective if it has good classification performance, which is measured by accuracy, sensitivity, and precision. Sensitivity is the percentage (or proportion) of dengue outbreaks (Yes) correctly classified by the model. Precision is the percentage (or proportion) of dengue outbreak cases classified correctly as a dengue outbreak (Yes). Overfitting problems occur when the testing values are much lower than the training values. This procedure also helps detect if there is an overfitting problem where the model performs well in the training sample but not in the testing sample.

CART, known as Classification and Regression Tree, is a decision tree model that uses Gini as a splitting criterion for a categorical target variable. The Artificial Neural network (ANN) model is an Artificial Intelligence model that consists of input layers, hidden layers, and output layers. The input layer data is connected to the hidden layers which have hidden neurons. Activation functions, such as the sigmoid function, are used to produce output values in the output layer. The ANN model allows modeling of a complex relationship between the input and output variables. The SVM is a machine learning classification model based on decision boundary and convex optimization problem, which can be solved using the Lagrange multiplier method**.** The linear kernel is used if the decision boundary (separated between the two classes of the target variable) is linear. The polynomial, sigmoid, and Gaussian Radial Basis kernel functions can be used when the decision boundary is nonlinear. The Naïve Bayes model is based on the Bayes Theorem of calculating the posterior probability of the event based on several attributes or independent variables^17,18^.

The models are similar in that they can obtain the probability for the binary target variable and identify the important predictors. They differ, however, in their methods of obtaining the model. Logistic regression and Naïve Bayes are both statistical methods, however the logistic regression model uses the Maximum Likelihood method for parameter estimation and logistic function while the Naïve Bayes method uses Bayes’ Theorem to calculate posterior probabilities. CART is a decision tree model that uses Gini as splitting criteria and provides decision rules, such as information on the relationship between input and target variable. The Support Vector Machine uses decision boundary and optimization theory to obtain the maximal linear and non-linear boundaries for binary classification problems.

The dengue fever outbreak graph was created using Tableau, a data visualization software. ArcGIS 10.7 was used for mapping.

### Ethical approval

Ethical approval was obtained from the Medical Research and Ethics Committee (MREC), Ministry of Health Malaysia (NMRR ID: NMRR-17-218-34011). The authors used de-identified delinked aggregated data and the requirement for consent has been waived off by approval of the Ethics committee. All methods were performed in accordance with the relevant guidelines and regulations.

## Results

**Figure 1:**
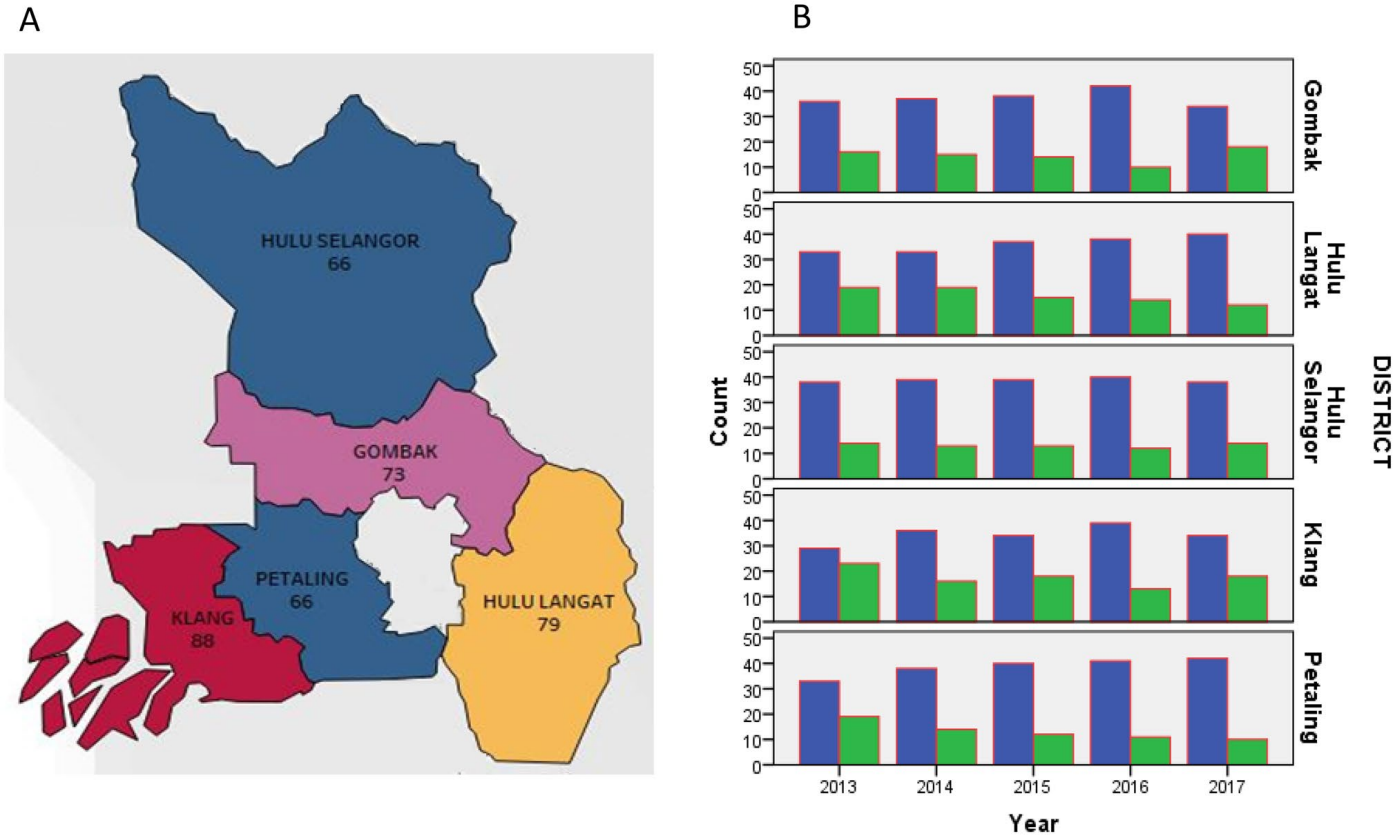
(**A**) Dengue fever outbreak in Selangor (study areas). ArcGIS 10.7 was used to create the map (https://desktop.arcgis.com/en/), (**B**) Dengue fever outbreaks by district and year (blue is representing no and green is representing yes).

Through analysis there were determined to be an increase in outbreaks in Gombak and Klang in 2017 (Table 4, supplement). Klang recorded the highest number of outbreaks from 2013 to 2017 (Fig. 1A). Results show that the Klang district had the highest number of dengue fever outbreaks, while Hulu Selangor had the least (Fig. 1B).

**Figure 2:**
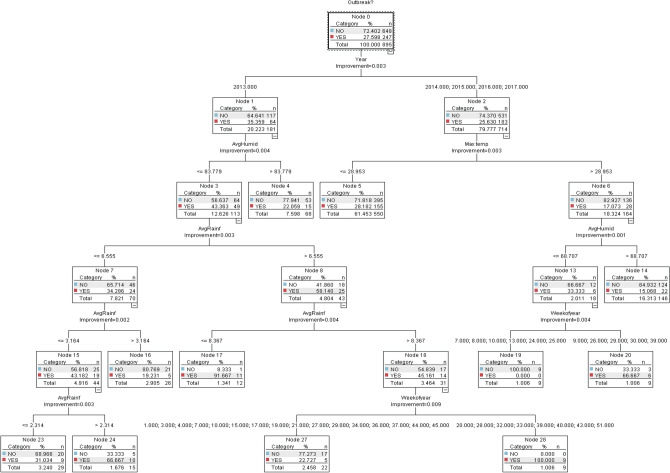
CART model.

**Figure 3:**
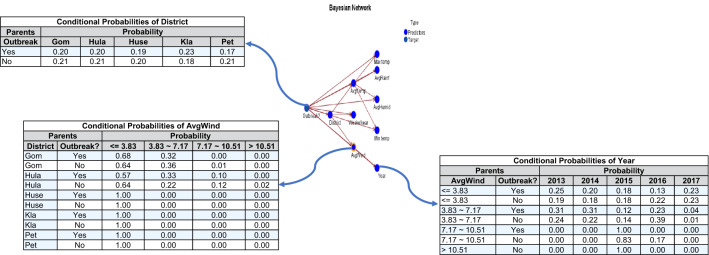
Naïve Bayes (TAN) model.

The visualization dashboard (Fig. 2, supplement) exhibits the district, as well as the week-of-year, in which the outbreaks occurred. By selecting the filters on the right-side panel, viewers can compare dengue fever outbreaks by district, year and outbreak status. In 2016, dengue fever incidence was highest in weeks 8 and 9, while maximum temperature peaked around week 16 in the Petaling district (Fig. 2, supplement). The early period of heavy rainfall in weeks 12 to 17 in Gombak is accompanied by more frequent outbreaks of dengue fever (indicated by the red bars), however that pattern does not continue throughout the year (Fig. 3, supplement). Outbreaks of dengue fever initially occurred in weeks 2, 6 and 7, with an increasing trend in wind speed in Hulu Langat followed by a sudden drop in week 43. This subsequently occurred within several weeks in the middle of the year and in the final quarter of 2015 (Fig. 4, supplement).

### Data mining techniques analysis

Based on the CART decision rules, no outbreaks occurred in 2013 (Fig. 2) when weekly humidity was more than 83.8 g/kg. However, when humidity was less than 83.8 g/kg, and rainfall was between 2.3 and 3.2 mm, outbreaks did occur. From 2014 to 2017, outbreaks occurred when humidity was less than 68.7 g/kg and maximum temperature was more than 28.95 °C.

Predictor importance for the decision tree (CART) model shows that maximum temperature is the most important variable for CART and ANN models, while Week of Year is the most important variable for SVM models (Table 1).

**Table 1:** Predictor importance of the models.

	Decision tree	Artificial neural network	SVM (linear)	SVM (polynomial)	SVM (RBF)	Naive Bayes
Week of year	0.13	0.22	0.50	0.20	0.73	0.10
Average temperature	0.70	0.19	0.06	0.04	0.03	0.13
Average humidity	0.22	0.07	0.06	0.03	0.01	0.11
Average wind	0.70	0.05	0.06	0.02	0.01	0.11
Maximum temperature	0.14	0.22	0.06	0.04	0.02	0.10
Minimum temperature	0.70	0.05	0.06	0.01	0.01	0.11
Average rainfall	0.10	0.07	0.06	0.03	0.01	0.11
Year	0.11	0.08	0.06	0.15	0.11	0.11
District	0.70	0.06	0.06	0.60	0.07	0.13

The machine learning algorithms used the variance based method to calculate the predictor importance. First the predictors are ranked according to the sensitivity measure using the following formula (IBM SPSS Modeler Algorithms Guide, 2016):

$${S}_{i}=\frac{{V}_{i}}{V\left(Y\right)}=\frac{V\left(E\left(Y|{X}_{I}\right)\right)}{V\left(Y\right)}$$

where V(Y) is the unconditional output variance. Predictor importance is then computed as the normalized sensitivity using the following formula:

$${VI}_{i}=\frac{{S}_{i}}{\sum_{j=1}^{k}{S}_{j}}$$

### Support vector machine (SVM)

All three SVM models (linear, polynomial and RBF) selected “week-of-year” as the most important predictor (Table 1). The results in Table 2 demonstrate that the Linear SVM model performed better than both the polynomial (degree = 2) and RBF kernels (Table 2). The SVM Polynomial and RBF models exhibit overfitting, as the accuracy, specificity and sensitivity results are very high for the training sample and low for testing sample. Overfitting occurs when a model classification performance is good for the training sample, but performs badly in the testing sample. The SVM Linear is chosen to be compared with CART, ANN and Naïve Bayes model.

**Table 2:** Model evaluation (performance comparison) results-original and balanced data.

	Sample	Accuracy (%)		Specificity (%)		Sensitivity (%)		Precision (%)	
		Original	Balanced	Original	Balanced	Original	Balanced	Original	Balanced
CART	Training	78.77	64.95	96.14	51.81	33.20	76.81	76.64	63.86
	Testing	63.21	50.23	86.07	37.19	12.00	66.67	27.78	45.71
SVM (LINEAR)	Training	75.31	68.38	96.76	63.45	19.03	72.83	69.12	68.84
	Testing	70.12	57.14	95.00	52.07	14.40	63.54	56.25	51.26
SVM (POLYNOMIAL)	Training	100	100	100	100	100	100	100	100
	Testing	65.43	53.76	77.86	48.76	37.60	64.58	43.12	50.00
SVM (RBF)	Training	86.82	86.10	94.44	84.34	66.80	87.68	82.09	86.12
	Testing	65.93	59.45	80.00	57.85	34.40	61.46	43.43	53.64
Naïve Bayes (TAN)	Training	82.94	82.10	92.74	78.31	57.33	85.51	75.14	81.38
	Testing	61.50	54.19	76.26	51.00	27.43	58.23	33.33	48.42
ANN (MLP)	Training	73.87	97.90	78.63	97.99	18.10	97.83	59. 15	98.18
	Testing	66.14	55.30	95.21	54.55	13.68	56.25	37.21	49.54

a = Original data (Yes: 372(28.62%), No = 928(71.38%), b = Balanced Data (Yes = 372(50.1%), No = 370 (49.9%)).

### Naïve Bayes

A Naïve Bayes prediction model was developed using Tree Augmented Naïve Bayes (TAN) structure. The model structure is depicted in Fig. 3. Using the model, suppose the necessity to classify case X (Gombak, 2014, wind speed = 2 m/s). Based on the conditional probability given in Fig. 3, the probability of an outbreak versus no outbreak occurring in Gombak in 2014, with a wind speed of 2 m/s, can be obtained as follows:

- P (outbreak = Yes |Gombak, wind speed = 2, year = 2014) = 0.20 × 0.68 × 0.21 = 0.029.
- P (outbreak = No |Gombak, wind speed = 2, year = 2014) = 0.21 × 0.64 × 0.20 = 0.027.

The probability of an outbreak to occur in Gombak in 2014, with a wind speed of 2 m/s, is higher (probability = 0.029) than the probability of an outbreak not occurring (probability = 0.027). Furthermore, case X would be classified (Gombak, 2014, wind speed = 2 m/s) as an Outbreak = Yes.

### Model evaluation

The models were evaluated based on classification accuracy, sensitivity, specificity, and precision. Based on the results presented (Tables 1 and 2), and the ROC curve (Fig. 5, supplement), overfitting occurs. Results in Table 2 shows that overfitting problems exists for the CART, SVM (Polynomial), SVM (RBF), Naïve Bayes and ANN models, as the models performed well in the training sample but not for the testing sample. Only the SVM Linear model performance was consistent for both training and testing samples. Based on testing sample results, the SVM Linear model (Accuracy = 70%, Sensitivity = 14%, Specificity = 95%, Precision = 56%) performed better than CART (Accuracy = 63%, Sensitivity = 12%, Specificity = 86%, Precision = 28%), Tree Augmented Naïve Bayes (Accuracy = 62%, Sensitivity = 27%, Specificity = 76%, Precision = 33%) and ANN (Accuracy = 66%, Sensitivity = 14%, Specificity = 95%, Precision = 37%). Results in Table 2 demonstrates all models over fit except for SVM (Linear), where the discrepancy between training and testing values is small.

The low sensitivity is due to the imbalance in the Dengue Outbreak variable. Out of 1300 cases, only 372 (28.62%) for Dengue Outbreak = Yes and 925 (71.38%) cases for Dengue Outbreak = No.

The low sensitivity is due to the imbalanced in the outbreak variable (Yes = 372 [28.6%], No = 928 [71.4%]). The imbalance will cause the performance of the classifiers to be biased toward the majority (Outbreak = No) samples. One simple way to overcome low sensitivity for imbalanced data is using the sampling strategy. We performed simple random under sampling^19^ and run the predictive modeling again for the new balanced data (Yes = 372 [50.1%], No = 370 [49.9%]).

In a random under-sampling approach, we keep all samples in the minority class and randomly selecting an equal (or almost equal) number of samples in the majority class, to obtain a balanced new dataset for further modeling.

The results for both original (imbalanced) and balanced data are shown in Table 2. The results for the balanced dengue data showed the sensitivity for all models has increased. All models still exhibit overfitting except for SVM (linear). The sensitivity for SVM (linear) for the testing sample is 63.54% compared to 14.4% for imbalanced (original data).

## Discussion

This study exemplifies that SVM, using a linear kernel, best predicted dengue outbreaks without overfitting. This is in agreement with other studies which utilized SVM to explore predictors of dengue fever^20–22^. This study serves to further validate SVM as a disease prediction tool and increase knowledge about its precision and accuracy.

Many studies have attempted to develop robust predictive models for worldwide dengue surveillance^21,23–26^. Authors found a linear model selected by the AIC stepdown method produced the strongest model for dengue prediction in Singapore. In previous studies Generalized linear models have been applied to dengue outbreak risk assessment. More recently, researchers have developed a real time model for predicting dengue in Singapore using a Least Absolute Shrinkage and Selection Operator (LASSO)^27^. The most state-of-the-art approach is the Support Vector Regression (SVR) model, which has been proven to be very effective with the time series prediction of dengue^21^. Although consideration was given to linear, polynomial, and RBF kernel for the support vector machine (SVM) model, the SVM linear model performed the best, as it does not exemplify overfitting. Because of this, the linear SVM model was selected for evaluation. The most commonly used statistical modeling techniques in dengue studies are Poisson Regression, Negative Binomial Regression^28^, Autoregressive Integrated Moving Average (ARIMA) and Generalized Additive Modeling (GAM). GAM^29^ and ARIMA^30^ are the standard reference models for associating environmental factors towards disease outcome and a tool for time series prediction analysis. In recent years, data driven techniques based on machine learning algorithms such as Decision Tree, Support Vector Machine, Naïve Bayes and Random Forest have shown promising results in predictive analytics for classification problems^31–33^.

As for individual predictors, the data visualization dashboards revealed a correlation between drastic peaks and rainfall with the increased number of dengue fever outbreaks. This corroborates what is known in the literature concerning both increased and decreased rainfall contributing to increased mosquito larva habitats^34^. Decreased rainfall could cause larger bodies of water to draw up into smaller pools that are suitable for mosquito larva survival^35^. Conversely, increased rainfall provides additional mosquito larva habitats in urban environments such as rainwater in old tires^36–38^.

Higher wind speeds correlated with lower dengue case counts, while moderate wind speeds correlated with higher numbers of dengue fever cases. It is conjectured that high wind speeds impede the *Aedes aegypti’s* ability to fly, and therefore limits host exposure to the vector^39^. Moderate wind speeds may contribute to increased interactions with hosts, and consequently more dengue infections^40^.

While some models selected maximum temperature as the most important predictor of dengue outbreaks, maximum temperature did not always correlate with the highest number of dengue outbreaks in the data dashboard. This is likely due to a lag effect, in which climate factors that impact both the mosquito lifecycle and viral replication take some time to ultimately impact vector-host interactions and infection rates^40^. Other works have found that increased temperature is positively correlated with dengue fever outbreaks with a lag of 0 to 3 weeks^40^. Population density and urbanization are also influential risk factor for the resurgence of dengue as reported by Struchiner et al.^41^.

Given the complexities of climate impact on host–vector–virus interactions, it is not surprising that the SVM model selected week-of-the-year as the most important predictor of dengue fever outbreaks. It could be that the humidity, wind speed, rainfall and temperature conditions that most favor viral replication in the vector as well as host–vector interactions occur around the same week each year. Week-of-the-year is also the most useful predictor of dengue outbreaks, as it contains interactions between the climate variables. For instance, the impact of humidity on dengue outbreaks may be dependent on temperature and/ or rainfall. This relationship could be captured more succinctly with the week-of-the-year variable rather than with complex ranges and combinations of climate variables. In this way, using week-of-the-year may help to simplify dengue early warning models at local scales and in locations with consistent seasonal climate conditions. Based on the CART model, patterns of dengue outbreak in 2013 were different from 2014 to 2017. In 2013, the dengue outbreak occurred in week 20, 28, 32, 22, 39, 40, and 51. Meanwhile, for 2014–2017, dengue outbreaks occurred in week 9, 26, 29, 30 and 39.

This study has several limitations, one of them being missing data. However, because no more than 15% of data was imputed, it is unlikely that missing data impacted findings^42^. Additionally, the low sensitivity is due to the imbalanced data in the target variable (Outbreak: Yes = 29%, No = 71%). Predictive modeling using a balanced sample improves the sensitivity of the models. All models still shows overfitting except for SVM (linear). Future studies can experiment with boosting algorithms or natured inspired algorithms (Particle Swarm Optimization or Grey Wolf) to increase the sensitivity of the model. The WHO outbreak definition specific to Malaysia was used, as Malaysia is a country in which dengue is an endemic disease. The study findings may not be generalizable to other countries due to meteorological elements. SVM cannot control the interactions between variables. Future work should include further investigation of SVM as an outbreak prediction tool as well as week-of-the-year as an important predictor of dengue outbreaks at different spatial scales as well as in different types of models. This method can be applied to predict other outbreaks of vector-borne diseases such as Chikungunya and Zika^43,44^.

## Conclusion

Machine Learning models are useful for classification and prediction of dengue fever outbreaks. This study created a new binary variable, dengue fever outbreak based on weekly dengue incidence data for Selangor and evaluated the performance of CART, ANN, SVM and Naïve Bayes model in the prediction of dengue outbreaks based on climate variables. The application of the machine learning models for prediction of dengue outbreak can provide vital information to healthcare authorities so that they can better prepare for dengue fever outbreaks. Examination of the week-of-the-year as the most important predictor of dengue outbreaks may simplify modelling and prevention efforts at local levels. Machine learning model has great potential for applications in epidemiology and disease outbreak studies.

## Supplementary Information

Supplementary Text.

Supplementary Figures.

Supplementary Tables.

## Data Availability

Data supporting the conclusions of this manuscript are provided within the article and will be available from the corresponding author upon request.
