# Supplementary material for: Prediction of dengue outbreak in Selangor Malaysia using machine learning techniques
Source: Sci Rep. 2021 Jan 13;11:939. doi: 10.1038/s41598-020-79193-2 (PMC7806812; doi:10.1038/s41598-020-79193-2)
Supplement: Supplementary file 1 — Supplementary Text. [file 41598_2020_79193_MOESM1_ESM.docx]

**Prediction of Dengue Outbreak in Selangor Malaysia using Machine Learning Techniques**

Supplement

Nurul Azam Mohd Salim^1^, Yap Bee Wah^2^, Caitlynn Reeves^3^, Madison Smith^3^, Wan Fairos Wan Yaacob^2^, Rose Nani Mudin^4^, Rahmat Dapari^4^, Nik Nur Fatin Fatihah Sapri^1^, Ubydul Haque^3^

^1^Advanced Analytics Engineering Centre, Faculty of Computer and Mathematical Sciences, Universiti Teknologi MARA 40450 Shah Alam, Selangor Malaysia.

^2^Faculty of Computer and Mathematical Sciences, Universiti Teknologi MARA Cawangan Kelantan, Kampus Kota Bharu, Lembah Sirrh, 15050 Kota Bharu, Kelantan, Malaysia.

^3^Department of Biostatistics and Epidemiology University of North Texas Health Science Center Fort Worth, TX 76107

^4^Vector Borne Disease Sector, Disease Control Division, Ministry of Health Malaysia, Level 4, Block E10, Complex E, Federal Government Administration Complex, 62590 Putrajaya Malaysia.

Methods

Dengue case data was extracted from the eDengue V2 surveillance system. The eDengueV2System provides accurate and real-time data for the district, state, and national levels. There are more than 350 variables in this system, including case management, prevention, control activities, outbreak management, source reduction activities, fogging, larviciding, ecosystem management, insecticide stockpile, and health education activities at the district and state level. Besides, providing real-time web-based surveillance, this system is also used to ensure prompt action can be taken on the prevention and control measures and comprehensive nationwide monitoring can be applied. This system is used only by the health staff, and has a security password for individual staff.

According to the World Health Organization (WHO)^1,2^, diagnosis may involve multiple detection methods depending on the dengue prognosis. To detect dengue in its earliest stages, individuals can be tested for the NS1 (non-structural 1) antigen. After illness onset, the virus can be detected in blood serum, plasma, blood cells, and other tissues for 4-5 days. During the acute stage of dengue, virus isolation and nucleic acid or antigen detection can be used for diagnosis. Thereafter, serological assays detecting IgM or IgG antibodies are the method of choice for diagnosis. First-time dengue infections typically have a stronger and more specific IgM response, whereas second-time infections demonstrate a weaker IgM response but a strong IgG response.

There are many studies demonstrating the high seroprevalence of dengue in Malaysia. One study^3^ indicated age is positively associated with dengue seropositivity with its highest prevalence (89.2%) in individuals between 45-54 years of age. Because the virus has four different serotypes, many individuals in Malaysia are left vulnerable to reinfection. Protection from one serotype does not give protection to all serotypes. The IgG rapid test kit is beneficial in that it can provide early diagnosis so that proper monitoring and control measures can be put into place to avoid dengue complications.

The Ministry of Health (MOH) Malaysia, through its expert committee, has made a decision to include in its policy and dengue program that testing with either NS1, IgM, IgG or PCR is compulsory in all suspected dengue cases in view to diagnose dengue cases earlier. MOH Malaysia committed annually to allocate their budget to ensure that the test is available at all levels. The objectives of performing IgG tests are to diagnose dengue, especially secondary infection, as early as possible and to provide appropriate treatment to prevent death.

In Malaysia, there are many rapid test kits which are apt to detecting positive IgG antibodies. The rapid test kit used by Ministry of Health Malaysia and supplied to all public health facilities have been verified by Institute of Medical Research of Malaysia and have a sensitivity and specificity of no less than 95%.

Data Preparation (Stage 3)

Data format errors were detected in the dataset. Identifying the relevant variables that may have effects on the incidence of the target variable is being performed in the variable selection phase. This step would be a straightforward process in a dataset with several independent variables. Variables that are not required in data modeling activity were removed. These variables are *‘Station number’, ‘Month’* and *‘Day’*. However, *‘Month’* and *‘Day’* are useful in generating the *‘date’* variable, which was used in generating the ‘*Week of year*’ variable for the climate dataset.

**Data Cleaning**

Data cleaning was performed by detecting and correcting the error and inaccurate values. The intensive cleaning was conducted using Microsoft Excel. Another step in the cleaning process was removing the inaccurate rows and all the typographical errors for creating a general representation of the value of a certain variable (for example ‘Hulu Langat’ within ‘District’ variable).

The missing value within the raw climate dataset was inconsistently recorded. The recoding method was different according to the recording station. While some were recorded as ‘-1.1’, or were recorded with ‘NA’, most of them were left with a blank record. Data imputation of these missing values was carried out using Climatological Mean of the Day (CMD) method in view of the available data at hand. It uses the average of the past daily value of the same day. Calculation of the estimated value (V_est_) is as follows:

$V_{\mathrm{est}}=\frac{\sum_{j=1}^{T} V_{ij}}{T}$

(4)

(6)

where V_i_ is the value of the variable for the i^th^ day of year j and T is the number of years data which are available^4^.

**Constructing New Variables**

The first variable which was constructed from the existing raw data is the *‘week of the year’* in the climate dataset. The new variable is necessary for the process of merging both datasets that were planned to be done later in the data preparation stage. The temporal climate dataset was three different attributes, namely *‘Year’, ‘Month’* and *‘Day’*. However, in fulfilling the intended data mining activity in this study, the climate data are required to have the ‘week of year*’* attribute. The new attribute was used as the referral ID in the process of merging the climate data with the weekly dengue fever case count data.  The ‘*week of year*’ variable was generated using the ‘*Year’*, *‘Month’* and *‘Day’* attributes (Supplement table 1 & 2).

The second and third newly constructed variables are the weekly ‘Maximum Temperature’ and ‘Minimum Temperature’. They were being generated in the climate dataset using the ‘temperature’ variables within the climate data. These variables are generated because several previous studies have found a significant relationship between the variables and the dengue outbreak^5-7^. The daily climate data were transformed into weekly data by taking the average of the daily value within a week. The missing value within a particular week-of-year is being ignored as the other available values are being taken to generate the average weekly value. For a week with missing value in all seven days, imputation using the Climatological Mean of the Day (CMD) method has been adopted. The averages of the imputed daily value for a given week were then used to generate the weekly value.

Model Evaluation

The calculation of sensitivity and specificity are based on the confusion matrix as follows:

|  | Predicted Y (Dengue Outbreak) | |
| --- | --- | --- |
| Actual Y  (Dengue Outbreak) | 0 (No) | 1(Yes) |
| 0(No) | TN | FP |
| 1(Yes) | FN | TP |

$$Accuracy= \frac{TN+TP}{TP+TN+FP+FN}$$

$$Sensitivity= \frac{TP}{TP+FN}$$

$$Specificity= \frac{TN}{TN+FP}$$

$$Precision= \frac{TP}{TP+FP}$$

Marked difference in testing and training demonstrates the model has overfitting problems (when classification performance is good for the training set but not for the testing set). Results in Suppl. Figure 4 shows that all the models, except SVM (Linear), have overfitting problems where the performance is very much lower for the testing set.

**Variables Transferred Automatically from eNotifikasi to eDengue system**

1. Notification Number

2. Notification Input Date

3. Notification Date

4. Facility Name

5. Types of Facilities

6. Facility Address

7. No MRN

8. Name of Ward

9. Hospital Admission Date

10. Onset Date

11. Date of Diagnosis

12. Diagnosis

13. Clinical Information

14. Name of Doctor

15. Doctor Tel. No

16. Patient Name

17. Identity Card / Passport No

18. Date of Birth

19. Nation

20. Country of Origin

21. Gender

22. Citizenship Status

23. Patient Status

24. Hospital Address

25. Hospital Tel. Number

26. Patient Occupation Category

27. Patient Work Place Address

28. Patient Work Place Tel. No

29. Name of Confirmation Test

30. Date of Sample Taken

31. Date of Sample Received by Laboratory

32. Test Results

33. Date of Results Released

34. Notes/ Comments

**Figure legends**

Supplement fig. 1. Primary Data Collection

Supplement fig. 2. Dengue Outbreak and Temperature in Study Areas (A. Gombak, B. Hulu Langat, C. Hulu Selangor D. Klang, E. Petaling). The yellow line on the left-hand side indicates average temperature. The red bar represents an outbreak within a week, whereas the blue bar represents no-outbreak.

Supplement fig. 3. Dengue Outbreak and Rainfall in Study Areas (A. Gombak, B. Hulu Langat, C. Hulu Selangor D. Klang, E. Petaling). The yellow line on the right-hand side indicates average rainfall. The red bar represents an outbreak within a week, whereas the blue bar represents no-outbreak.

Supplement fig. 4. Dengue Outbreak and Wind Speed in Study Areas (A. Gombak, B. Hulu Langat, C. Hulu Selangor D. Klang, E. Petaling). The yellow line on the right-hand side indicates average wind. The red bar represents an outbreak within a week, whereas the blue bar represents no-outbreak.

Supplement fig. 5. A. ROC chart of CART Decision Tree model, B. ROC chart of ANN (MLP) model, C. ROC chart of SVM models, D. ROC chart of Naïve Bayes (TAN) model, E. ROC chart of best model candidates

**References**

1 WHO. Enhancing dengue diagnosis and case management. Available at [<https://www.who.int/activities/enhancing-dengue-diagnosis-and-case-management>], last accessed 10.23.2020.

2 IPCC, 2013: Annex I: Atlas of Global and Regional Climate Projections [van Oldenborgh, G.J., M. Collins, J. Arblaster, J.H. Christensen, J. Marotzke, S.B. Power, M. Rummukainen and T. Zhou (eds.)]. In: Climate Change 2013: The Physical Science Basis. Contribution of Working Group I to the Fifth Assessment Report of the Intergovernmental Panel on Climate Change [Stocker, T.F., D. Qin, G.-K. Plattner, M. Tignor, S.K. Allen, V. Bex and P.M. Midgley (eds.)]. Cambridge University Press, Cambridge, United Kingdom and New York, NY, USA.

3 Dhanoa, A. *et al.* Seroprevalence of dengue among healthy adults in a rural community in Southern Malaysia: a pilot study. *Infect Dis Poverty* **7**, 1, doi:10.1186/s40249-017-0384-1 (2018).

4 Balachandrudu, N., Timothy, D., Michael, K.T. Optimal Estimation of the Climatological Mean. Journal of Climate, 2009, vol. 22, issue 18, p. 4845.

5 Ahmad, R. *et al.* Factors determining dengue outbreak in Malaysia. *PLoS One* **13**, e0193326, doi:10.1371/journal.pone.0193326 (2018).

6 Chowell, G. & Sanchez, F. Climate-based descriptive models of dengue fever: the 2002 epidemic in Colima, Mexico. *J Environ Health* **68**, 40-44, 55 (2006).

7 Diana, N., Tarmizi, A., Jamaluddin, F., Bakar, A. A., Othman, Z. A., Diana, N., Hamdan, A. R. (2013). Classification of Dengue Outbreak Using Data Mining Models. Research Notes in Inforation Science, 12(April), 71–75.
