# Supplementary material for: Prediction of dengue outbreak in Selangor Malaysia using machine learning techniques
Source: Sci Rep. 2021 Jan 13;11:939. doi: 10.1038/s41598-020-79193-2 (PMC7806812; doi:10.1038/s41598-020-79193-2)
Supplement: Supplementary file 2 — Supplementary Figures. [file 41598_2020_79193_MOESM2_ESM.pptx]

## Slide 1
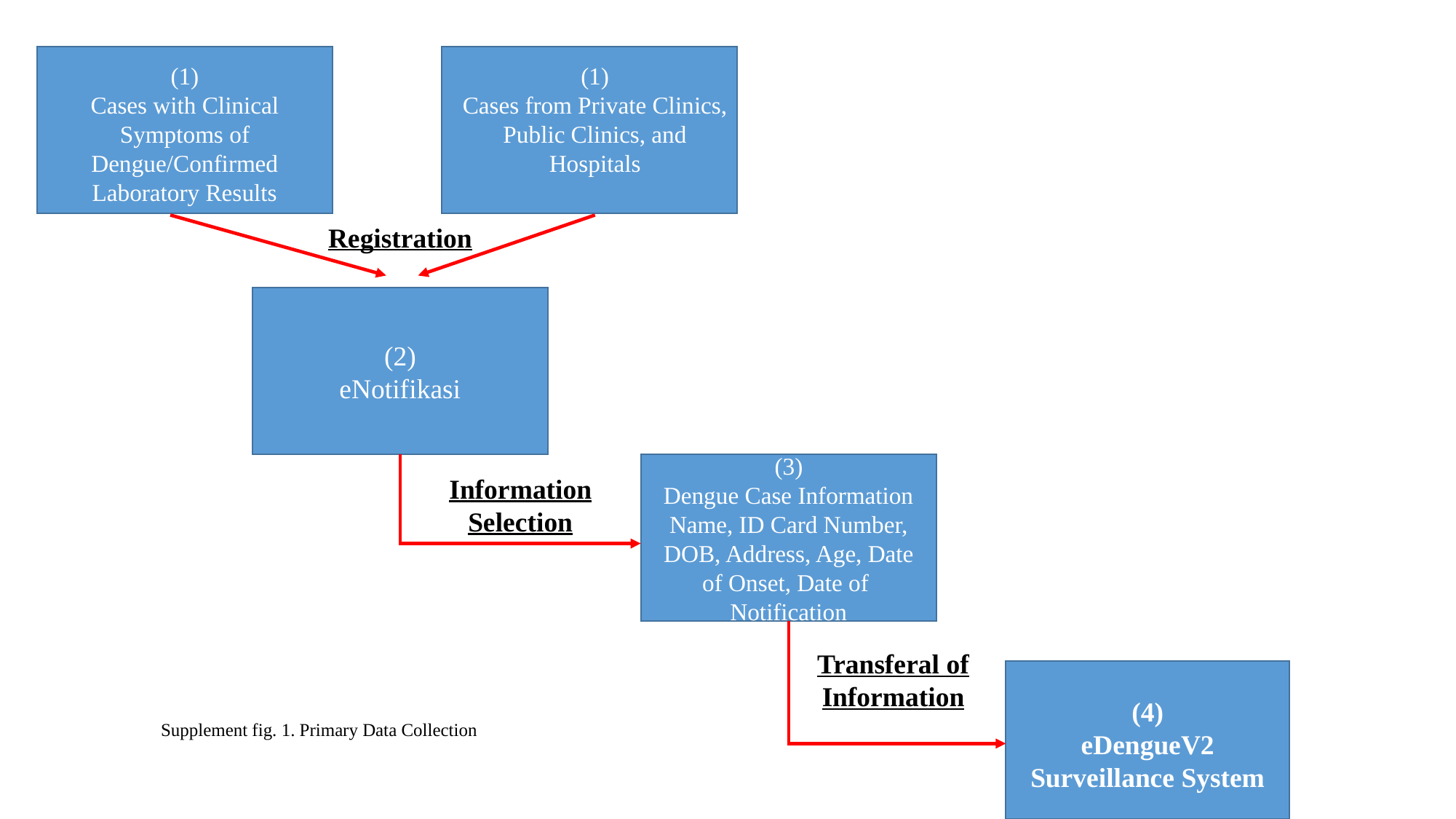

(1)
Cases with Clinical Symptoms of Dengue/Confirmed Laboratory Results
(1)
Cases from Private Clinics, Public Clinics, and Hospitals
Registration
(2)
eNotifikasi
(3)
Dengue Case Information Name, ID Card Number, DOB, Address, Age, Date of Onset, Date of Notification
Information Selection
Transferal of Information
(4)
eDengueV2 Surveillance System
Supplement fig. 1. Primary Data Collection

## Slide 2
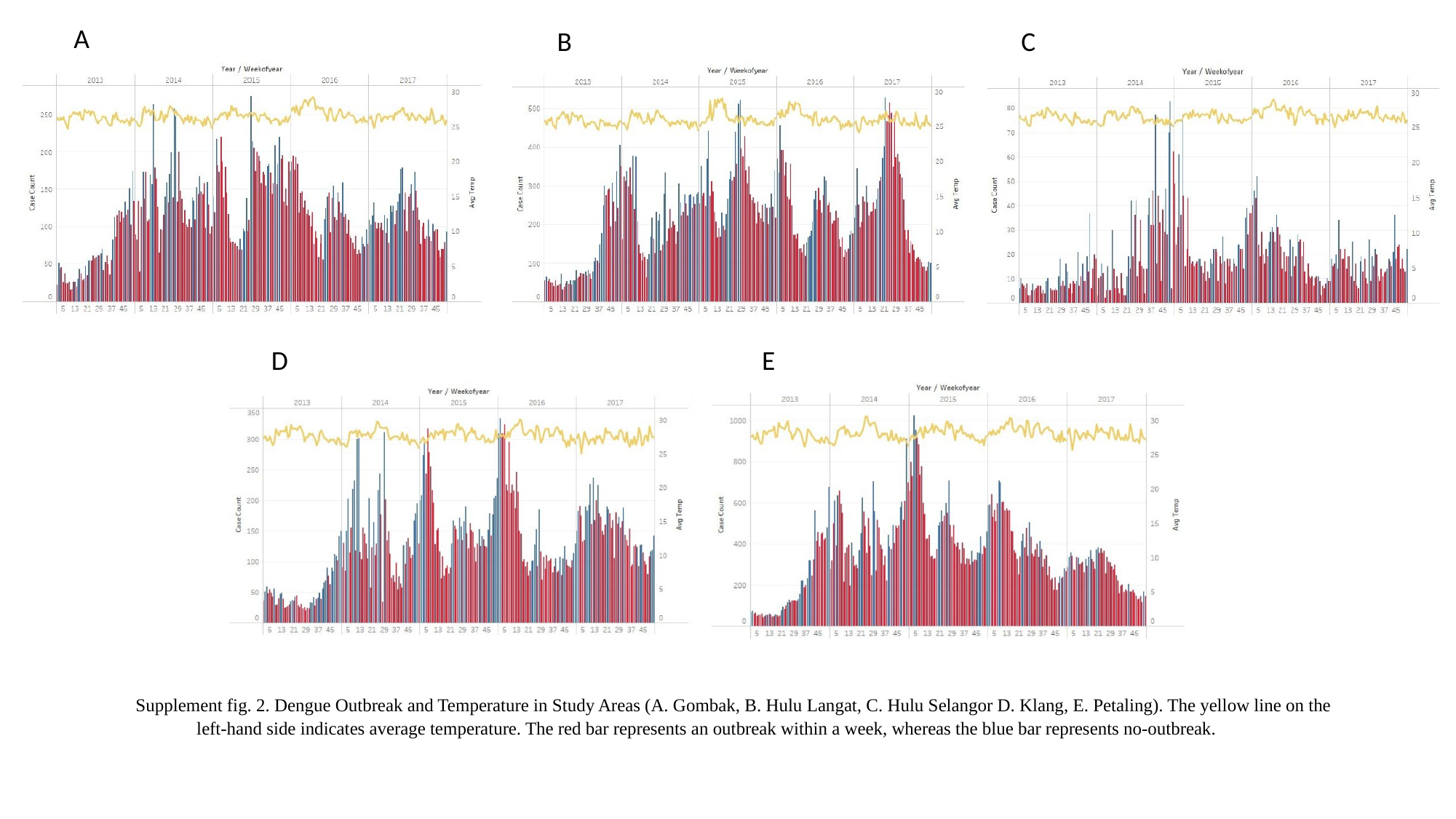

A
B
C
D
E
Supplement fig. 2. Dengue Outbreak and Temperature in Study Areas (A. Gombak, B. Hulu Langat, C. Hulu Selangor D. Klang, E. Petaling). The yellow line on the left-hand side indicates average temperature. The red bar represents an outbreak within a week, whereas the blue bar represents no-outbreak.

## Slide 3
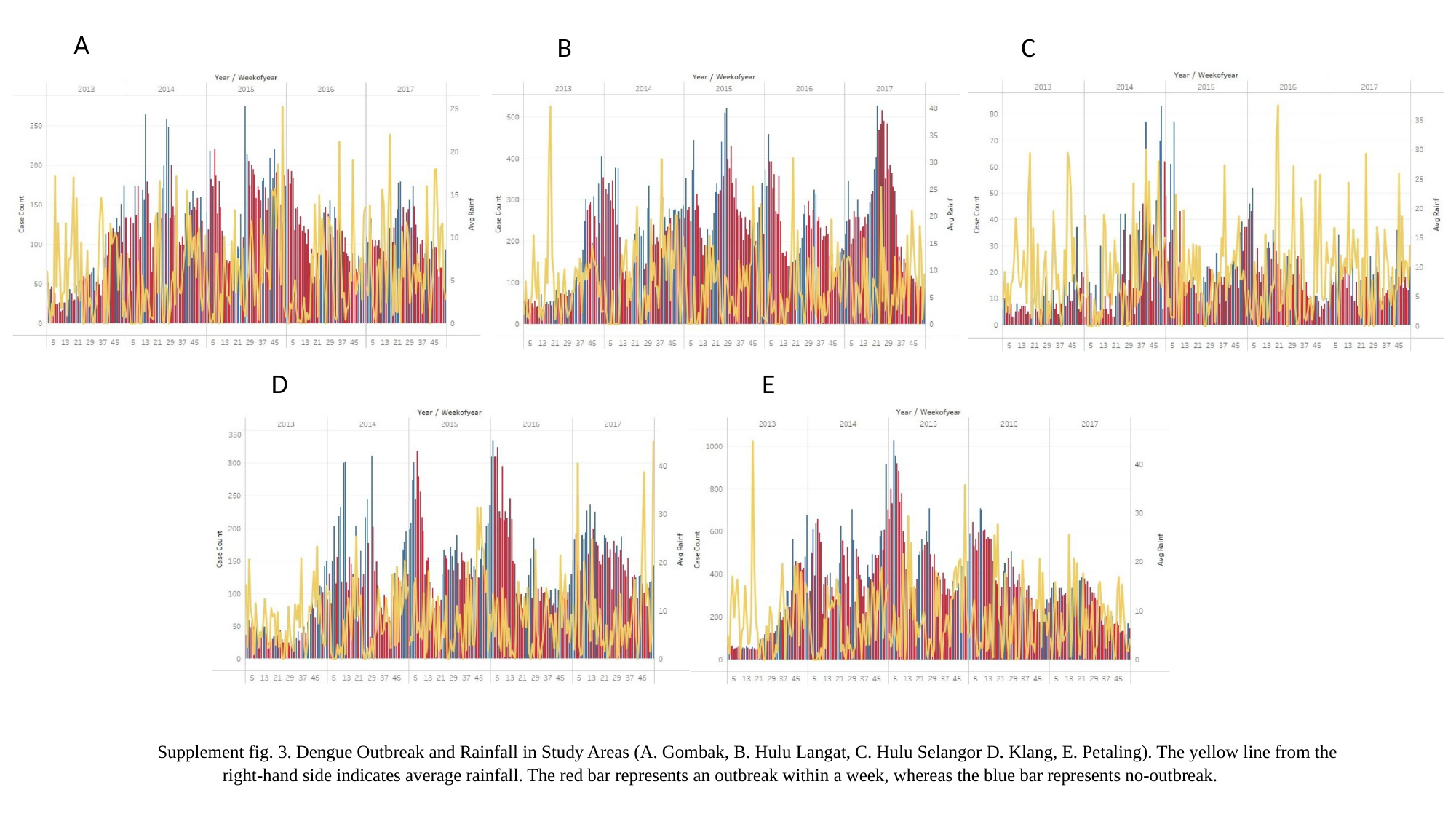

A
B
C
D
E
Supplement fig. 3. Dengue Outbreak and Rainfall in Study Areas (A. Gombak, B. Hulu Langat, C. Hulu Selangor D. Klang, E. Petaling). The yellow line from the right-hand side indicates average rainfall. The red bar represents an outbreak within a week, whereas the blue bar represents no-outbreak.

## Slide 4
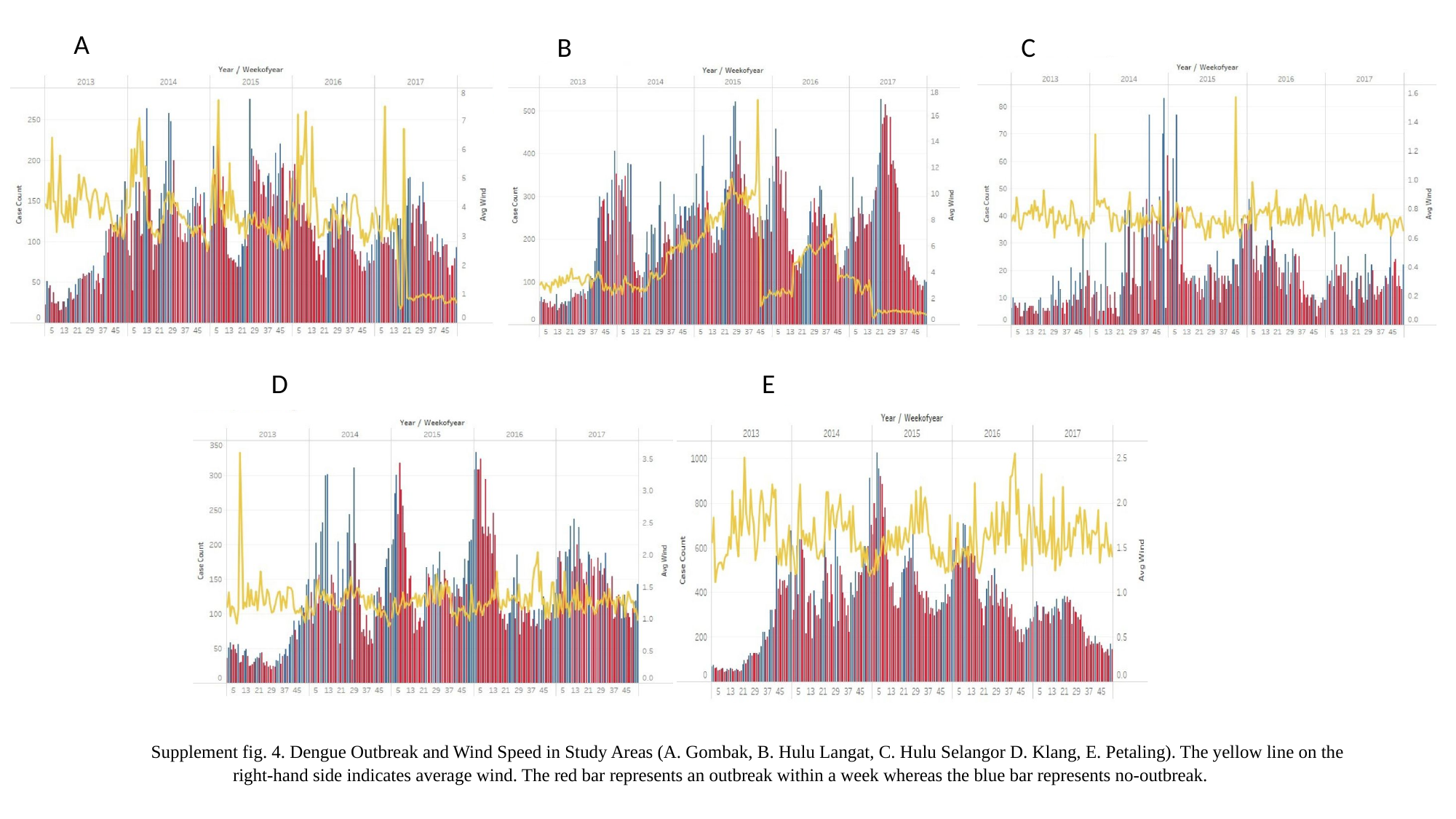

A
B
C
D
E
Supplement fig. 4. Dengue Outbreak and Wind Speed in Study Areas (A. Gombak, B. Hulu Langat, C. Hulu Selangor D. Klang, E. Petaling). The yellow line on the right-hand side indicates average wind. The red bar represents an outbreak within a week whereas the blue bar represents no-outbreak.

## Slide 5
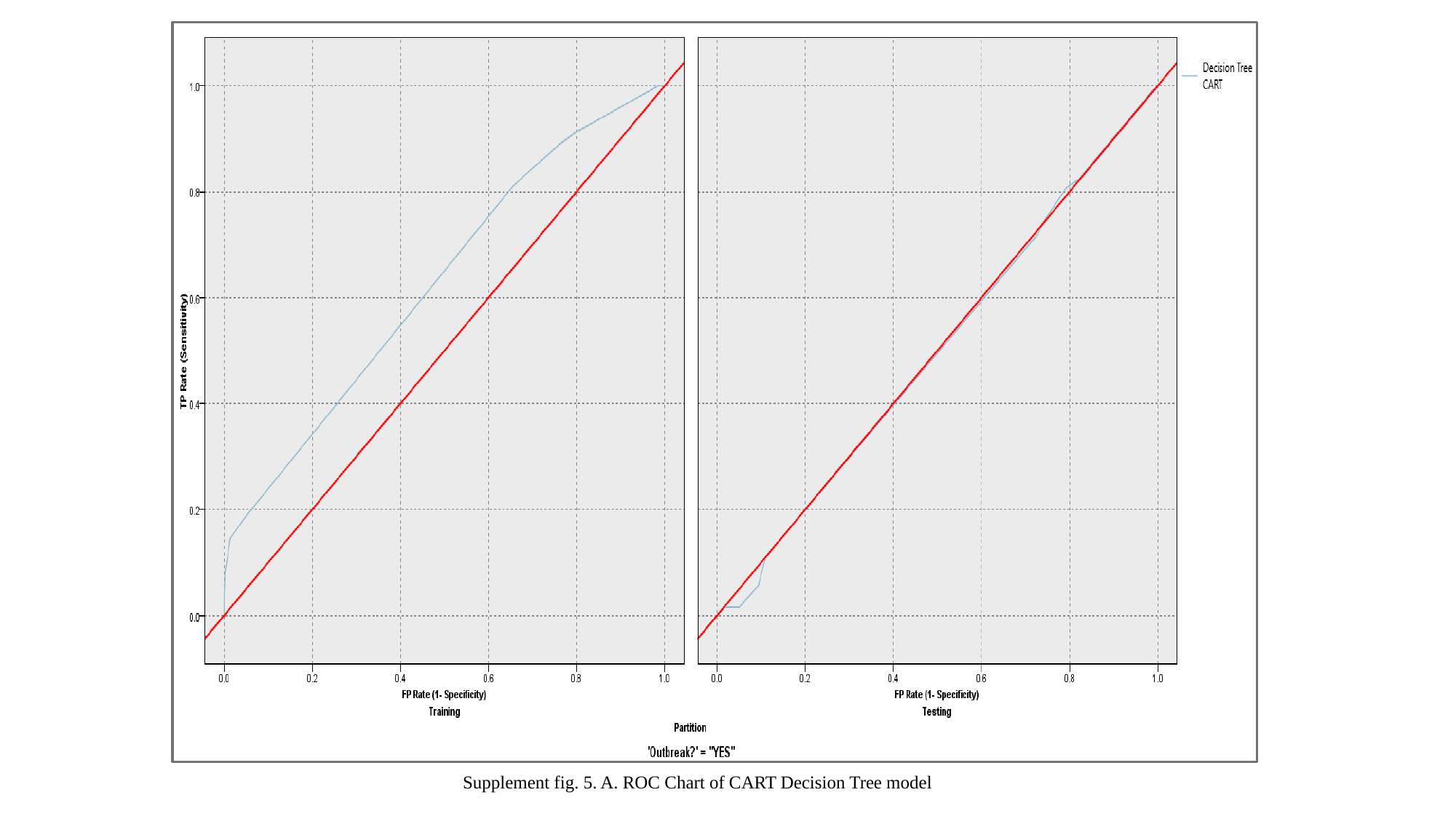

Supplement fig. 5. A. ROC Chart of CART Decision Tree model

## Slide 6
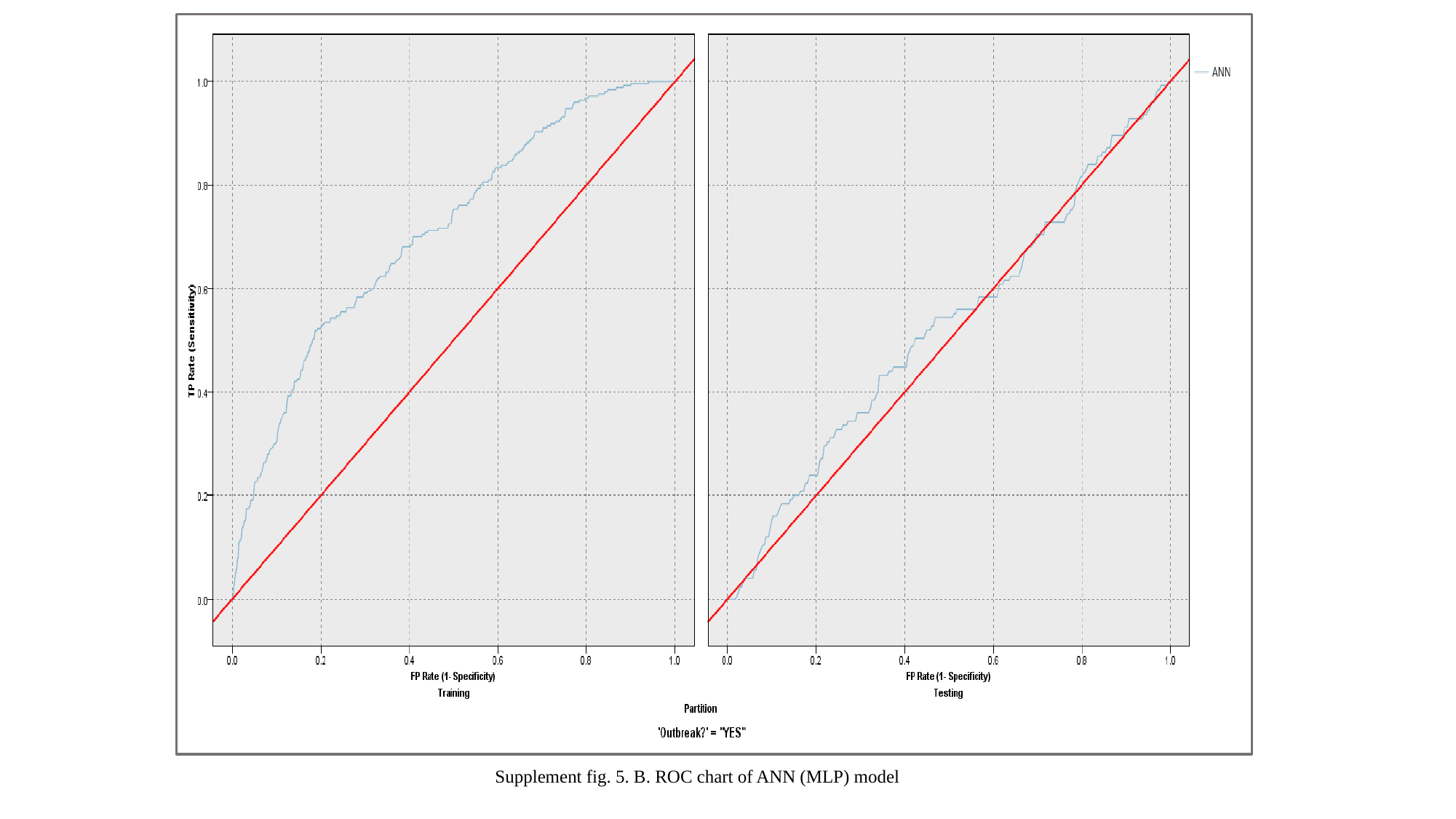

Supplement fig. 5. B. ROC chart of ANN (MLP) model

## Slide 7
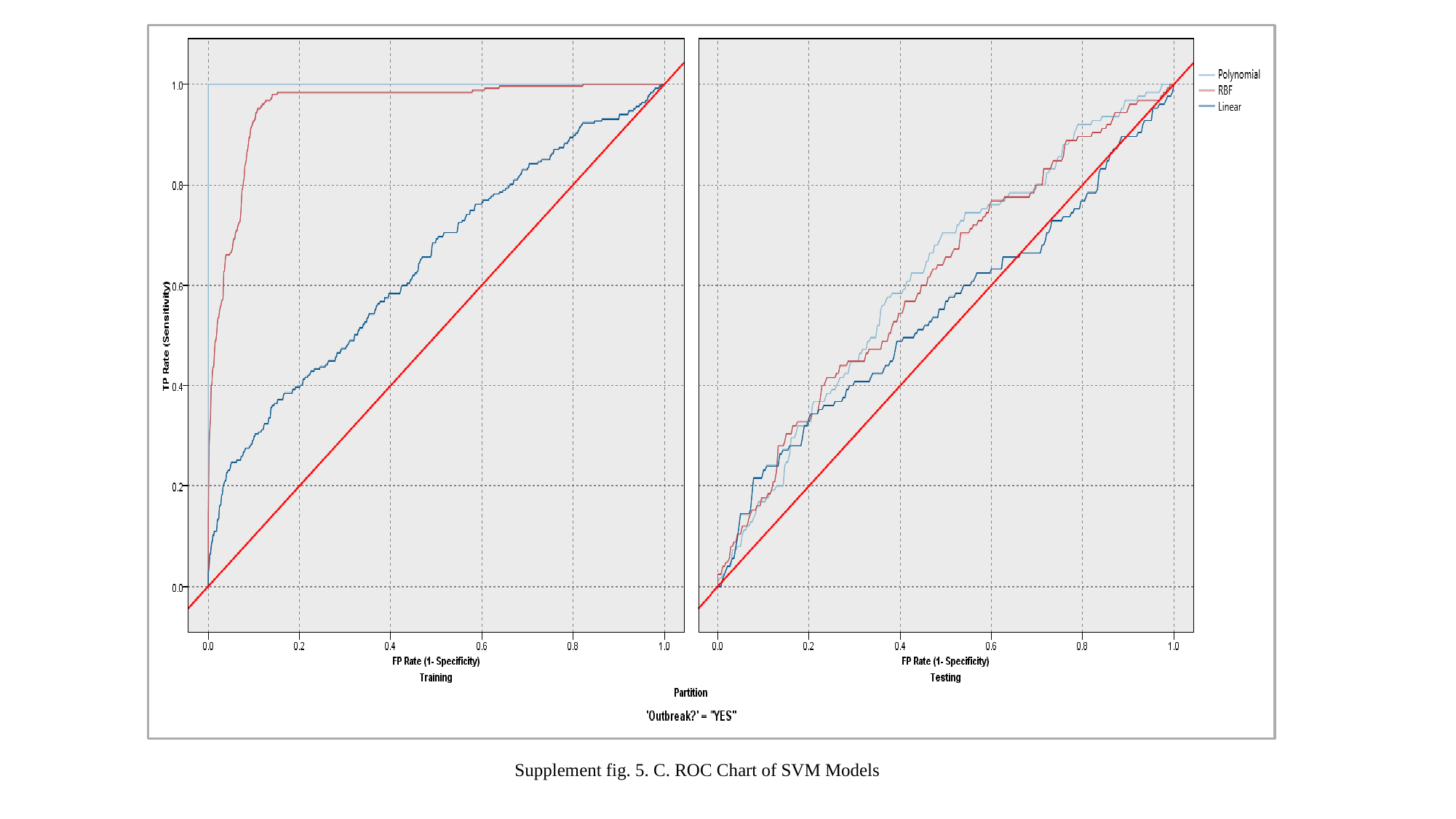

Supplement fig. 5. C. ROC Chart of SVM Models

## Slide 8
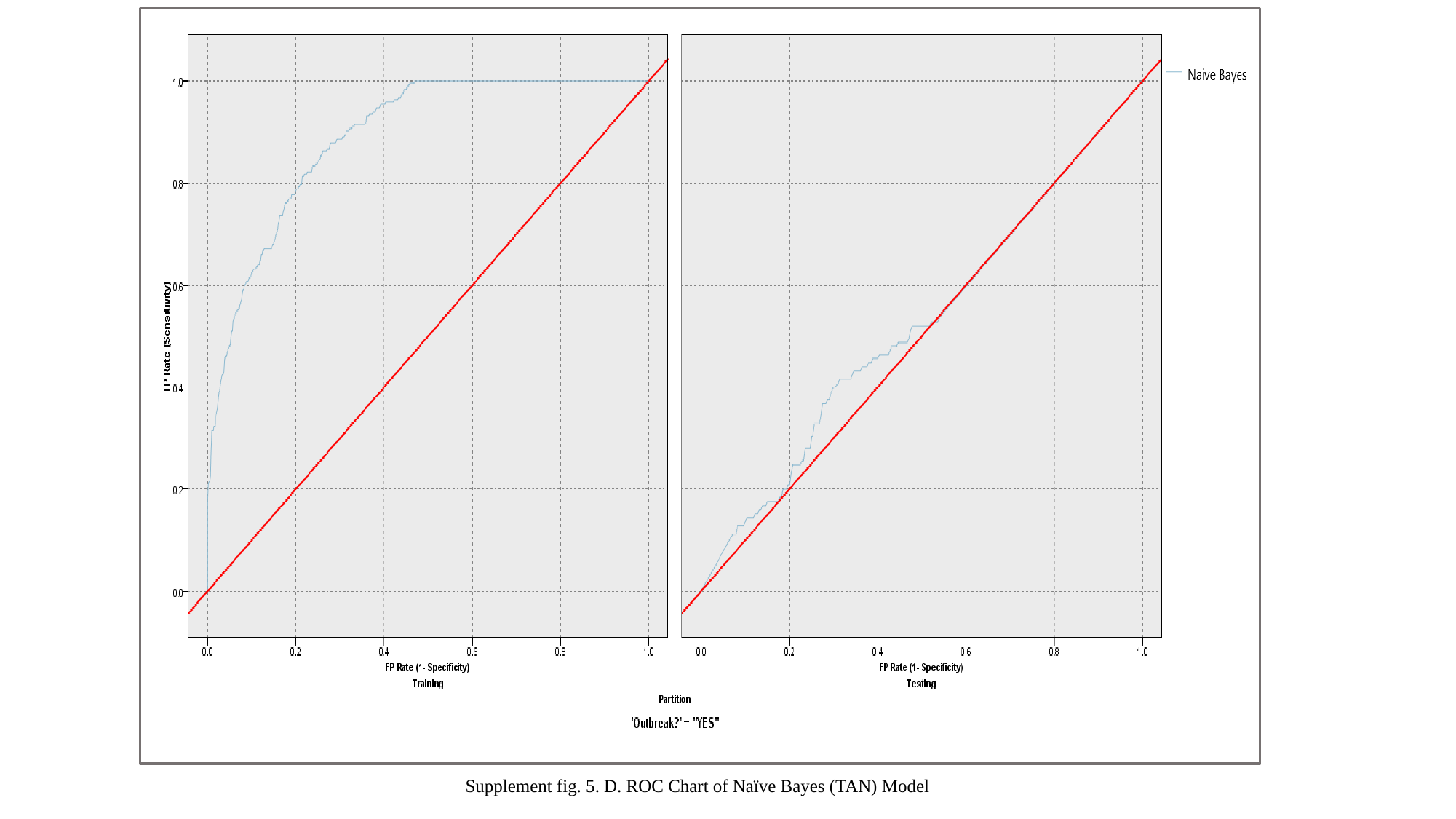

Supplement fig. 5. D. ROC Chart of Naïve Bayes (TAN) Model

## Slide 9
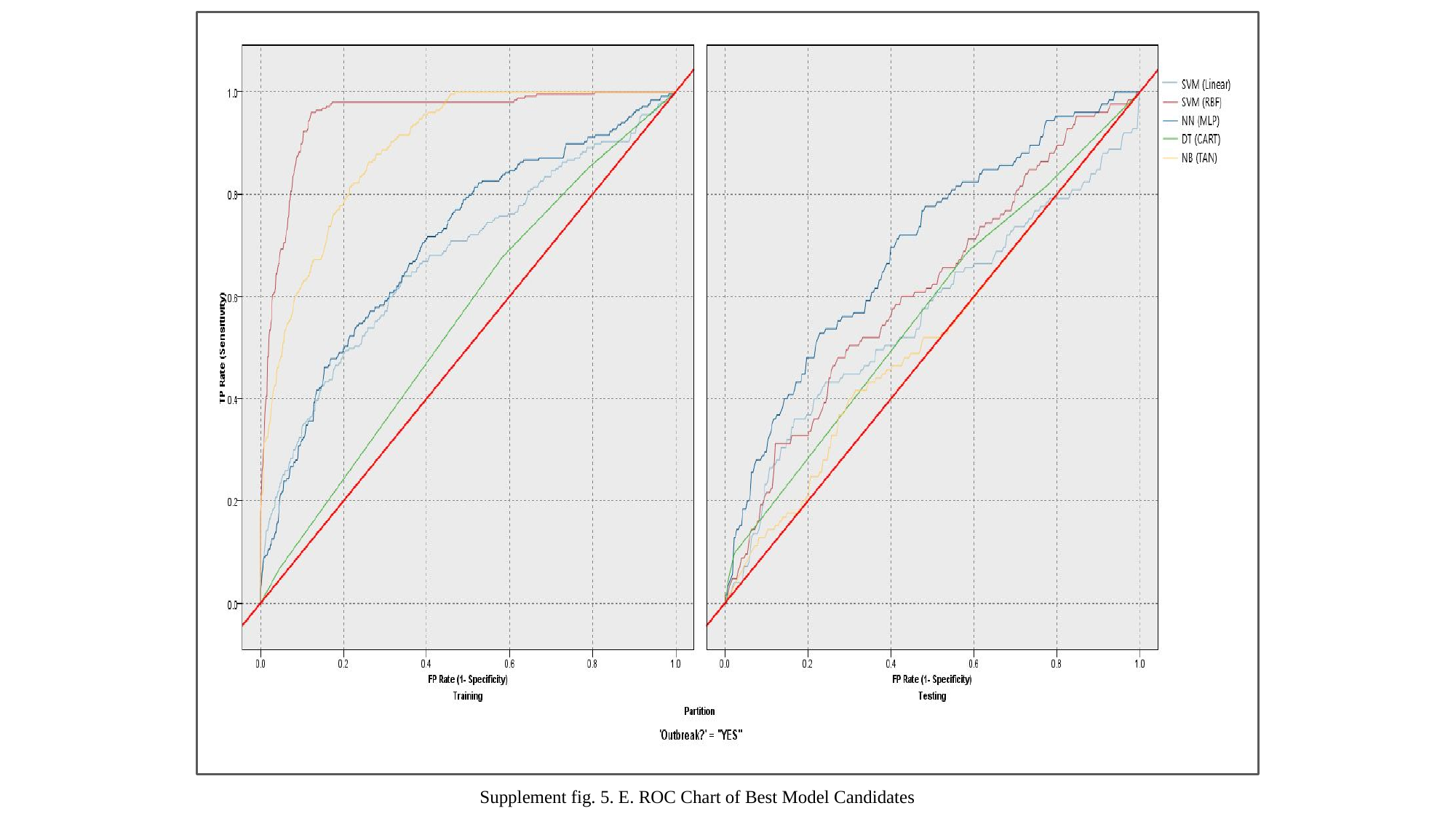

Supplement fig. 5. E. ROC Chart of Best Model Candidates
