# Supplementary material for: Prediction of dengue outbreak in Selangor Malaysia using machine learning techniques
Source: Sci Rep. 2021 Jan 13;11:939. doi: 10.1038/s41598-020-79193-2 (PMC7806812; doi:10.1038/s41598-020-79193-2)
Supplement: Supplementary file 3 — Supplementary Tables. [file 41598_2020_79193_MOESM3_ESM.pptx]

## Slide 1
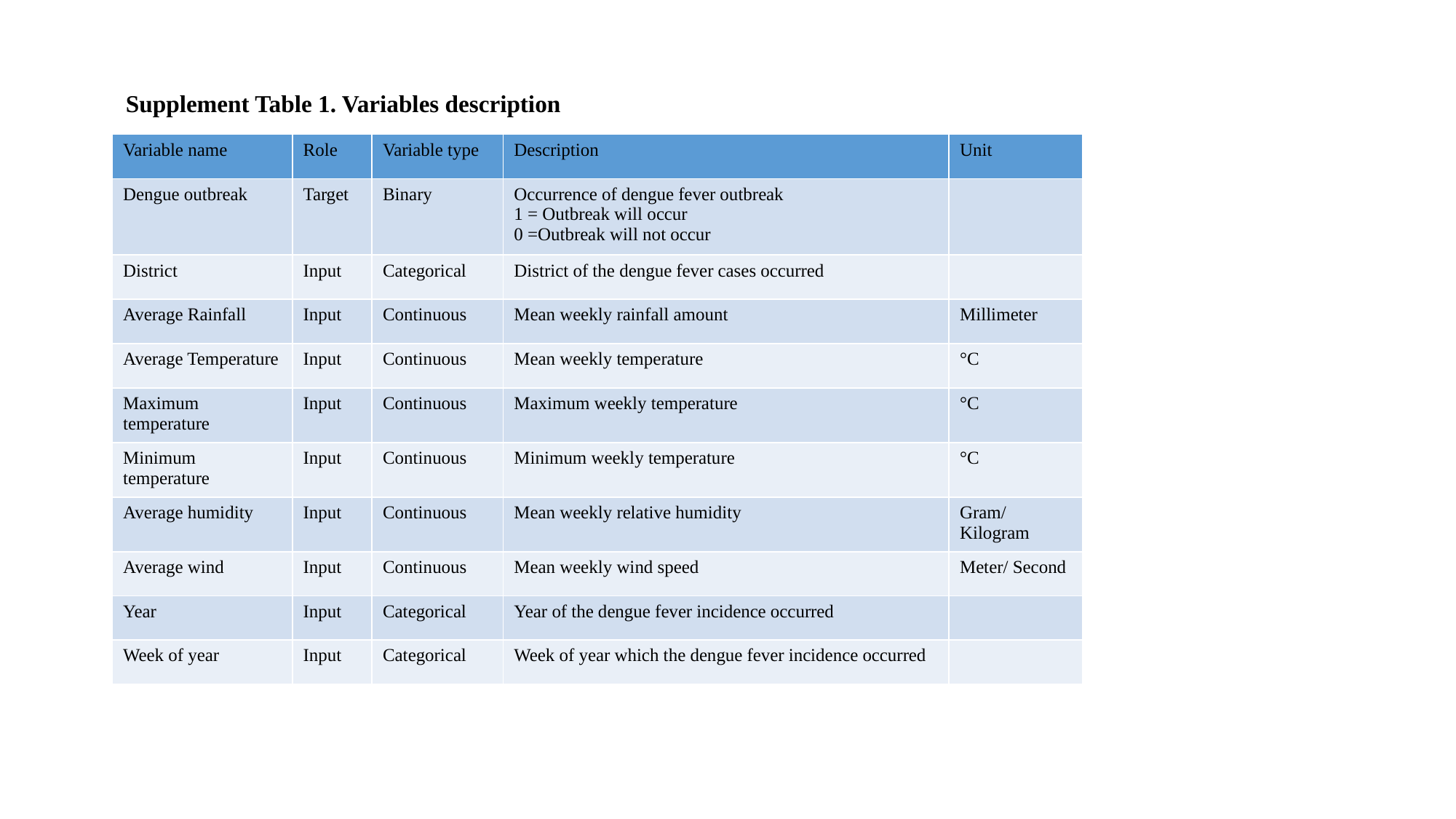

Supplement Table 1. Variables description
| Variable name | Role | Variable type | Description | Unit |
| --- | --- | --- | --- | --- |
| Dengue outbreak | Target | Binary | Occurrence of dengue fever outbreak 1 = Outbreak will occur 0 =Outbreak will not occur | |
| District | Input | Categorical | District of the dengue fever cases occurred | |
| Average Rainfall | Input | Continuous | Mean weekly rainfall amount | Millimeter |
| Average Temperature | Input | Continuous | Mean weekly temperature | °C |
| Maximum temperature | Input | Continuous | Maximum weekly temperature | °C |
| Minimum temperature | Input | Continuous | Minimum weekly temperature | °C |
| Average humidity | Input | Continuous | Mean weekly relative humidity | Gram/ Kilogram |
| Average wind | Input | Continuous | Mean weekly wind speed | Meter/ Second |
| Year | Input | Categorical | Year of the dengue fever incidence occurred | |
| Week of year | Input | Categorical | Week of year which the dengue fever incidence occurred | |

## Slide 2
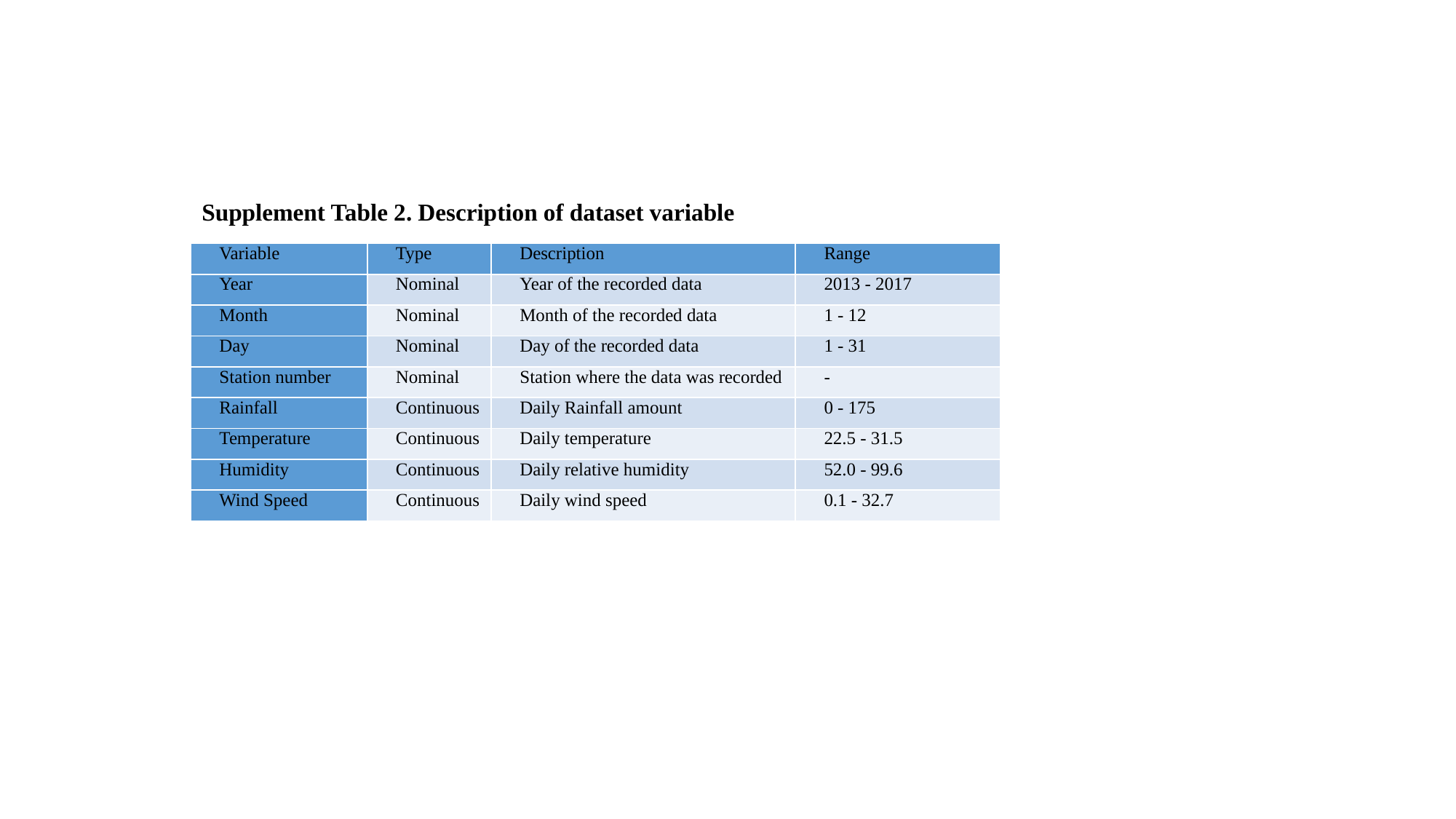

Supplement Table 2. Description of dataset variable
| Variable | Type | Description | Range |
| --- | --- | --- | --- |
| Year | Nominal | Year of the recorded data | 2013 - 2017 |
| Month | Nominal | Month of the recorded data | 1 - 12 |
| Day | Nominal | Day of the recorded data | 1 - 31 |
| Station number | Nominal | Station where the data was recorded | - |
| Rainfall | Continuous | Daily Rainfall amount | 0 - 175 |
| Temperature | Continuous | Daily temperature | 22.5 - 31.5 |
| Humidity | Continuous | Daily relative humidity | 52.0 - 99.6 |
| Wind Speed | Continuous | Daily wind speed | 0.1 - 32.7 |

## Slide 3
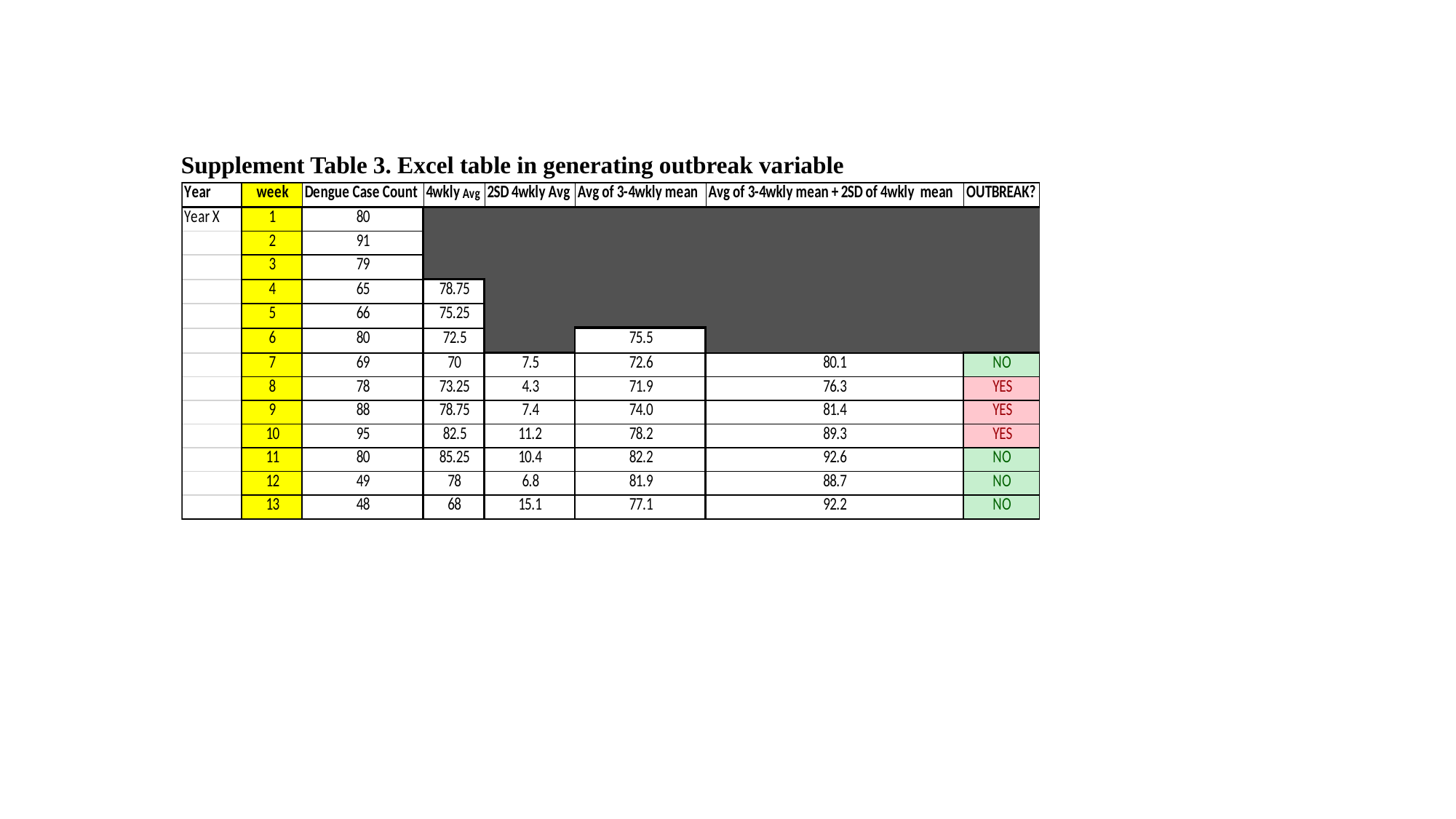

Supplement Table 3. Excel table in generating outbreak variable

## Slide 4
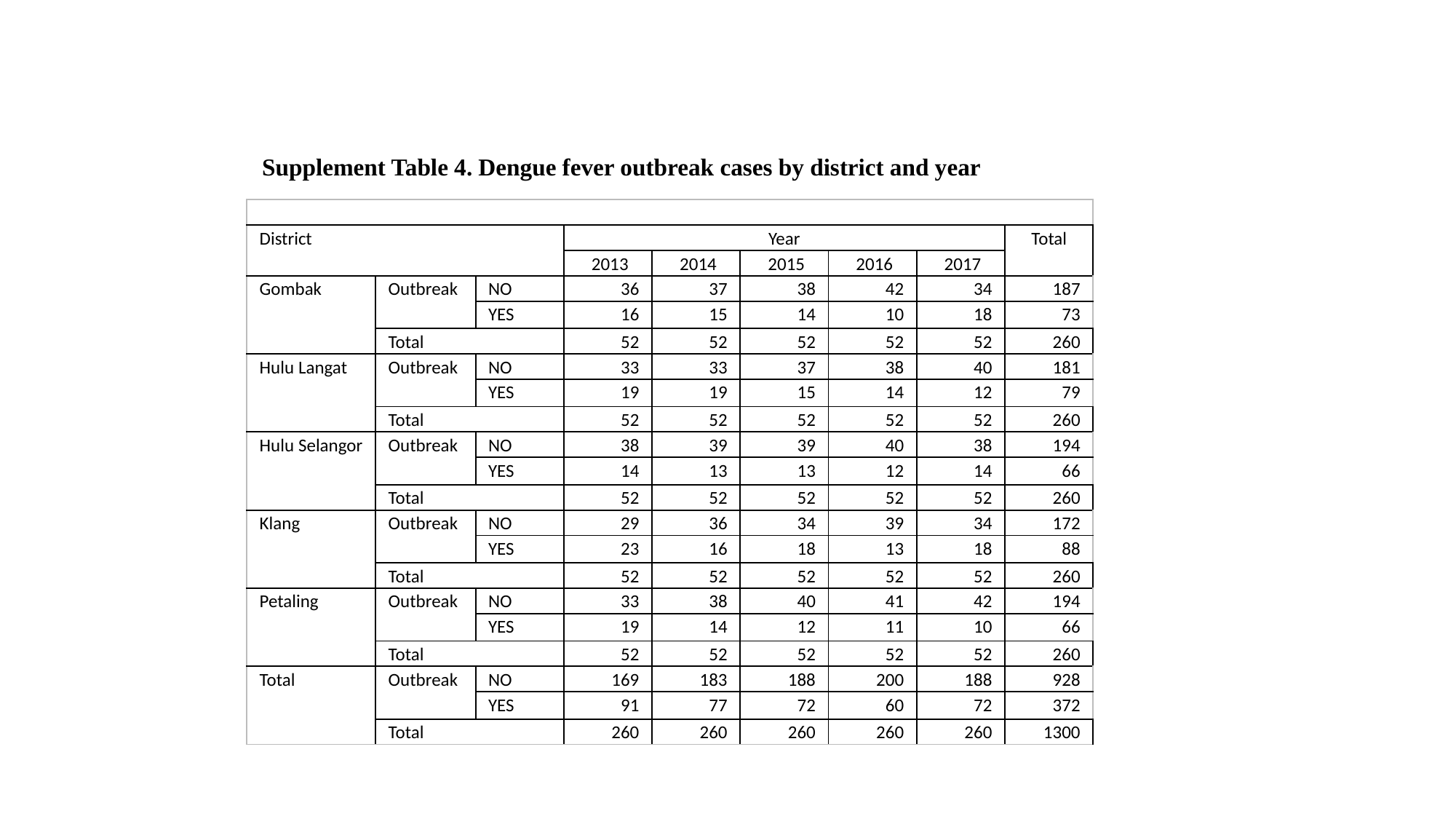

Supplement Table 4. Dengue fever outbreak cases by district and year
| | | | | | | | | |
| --- | --- | --- | --- | --- | --- | --- | --- | --- |
| District | | | Year | | | | | Total |
| | | | 2013 | 2014 | 2015 | 2016 | 2017 | |
| Gombak | Outbreak | NO | 36 | 37 | 38 | 42 | 34 | 187 |
| | | YES | 16 | 15 | 14 | 10 | 18 | 73 |
| | Total | | 52 | 52 | 52 | 52 | 52 | 260 |
| Hulu Langat | Outbreak | NO | 33 | 33 | 37 | 38 | 40 | 181 |
| | | YES | 19 | 19 | 15 | 14 | 12 | 79 |
| | Total | | 52 | 52 | 52 | 52 | 52 | 260 |
| Hulu Selangor | Outbreak | NO | 38 | 39 | 39 | 40 | 38 | 194 |
| | | YES | 14 | 13 | 13 | 12 | 14 | 66 |
| | Total | | 52 | 52 | 52 | 52 | 52 | 260 |
| Klang | Outbreak | NO | 29 | 36 | 34 | 39 | 34 | 172 |
| | | YES | 23 | 16 | 18 | 13 | 18 | 88 |
| | Total | | 52 | 52 | 52 | 52 | 52 | 260 |
| Petaling | Outbreak | NO | 33 | 38 | 40 | 41 | 42 | 194 |
| | | YES | 19 | 14 | 12 | 11 | 10 | 66 |
| | Total | | 52 | 52 | 52 | 52 | 52 | 260 |
| Total | Outbreak | NO | 169 | 183 | 188 | 200 | 188 | 928 |
| | | YES | 91 | 77 | 72 | 60 | 72 | 372 |
| | Total | | 260 | 260 | 260 | 260 | 260 | 1300 |

## Slide 5
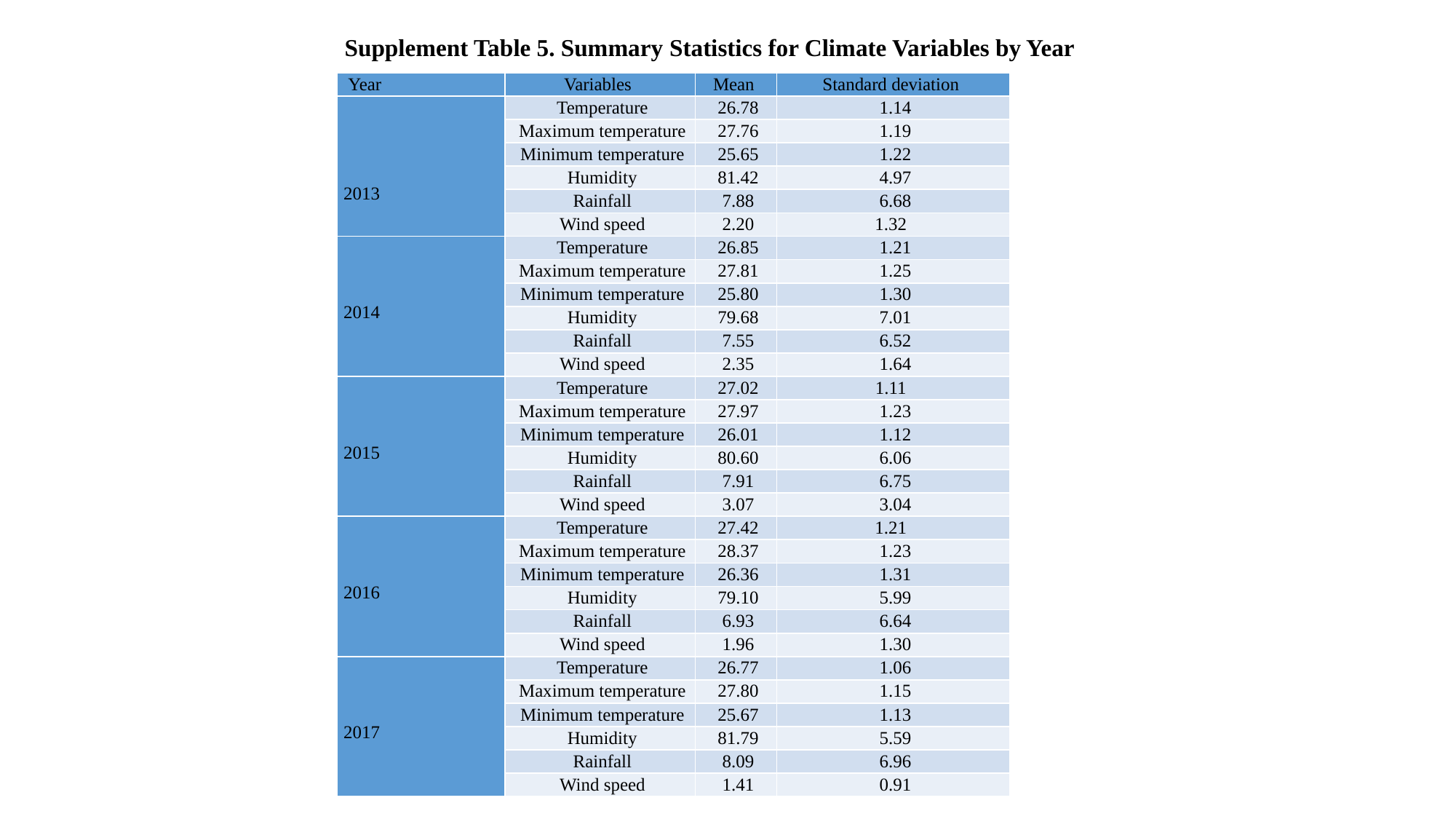

Supplement Table 5. Summary Statistics for Climate Variables by Year
| Year | Variables | Mean | Standard deviation |
| --- | --- | --- | --- |
| 2013 | Temperature | 26.78 | 1.14 |
| | Maximum temperature | 27.76 | 1.19 |
| | Minimum temperature | 25.65 | 1.22 |
| | Humidity | 81.42 | 4.97 |
| | Rainfall | 7.88 | 6.68 |
| | Wind speed | 2.20 | 1.32 |
| 2014 | Temperature | 26.85 | 1.21 |
| | Maximum temperature | 27.81 | 1.25 |
| | Minimum temperature | 25.80 | 1.30 |
| | Humidity | 79.68 | 7.01 |
| | Rainfall | 7.55 | 6.52 |
| | Wind speed | 2.35 | 1.64 |
| 2015 | Temperature | 27.02 | 1.11 |
| | Maximum temperature | 27.97 | 1.23 |
| | Minimum temperature | 26.01 | 1.12 |
| | Humidity | 80.60 | 6.06 |
| | Rainfall | 7.91 | 6.75 |
| | Wind speed | 3.07 | 3.04 |
| 2016 | Temperature | 27.42 | 1.21 |
| | Maximum temperature | 28.37 | 1.23 |
| | Minimum temperature | 26.36 | 1.31 |
| | Humidity | 79.10 | 5.99 |
| | Rainfall | 6.93 | 6.64 |
| | Wind speed | 1.96 | 1.30 |
| 2017 | Temperature | 26.77 | 1.06 |
| | Maximum temperature | 27.80 | 1.15 |
| | Minimum temperature | 25.67 | 1.13 |
| | Humidity | 81.79 | 5.59 |
| | Rainfall | 8.09 | 6.96 |
| | Wind speed | 1.41 | 0.91 |

## Slide 6
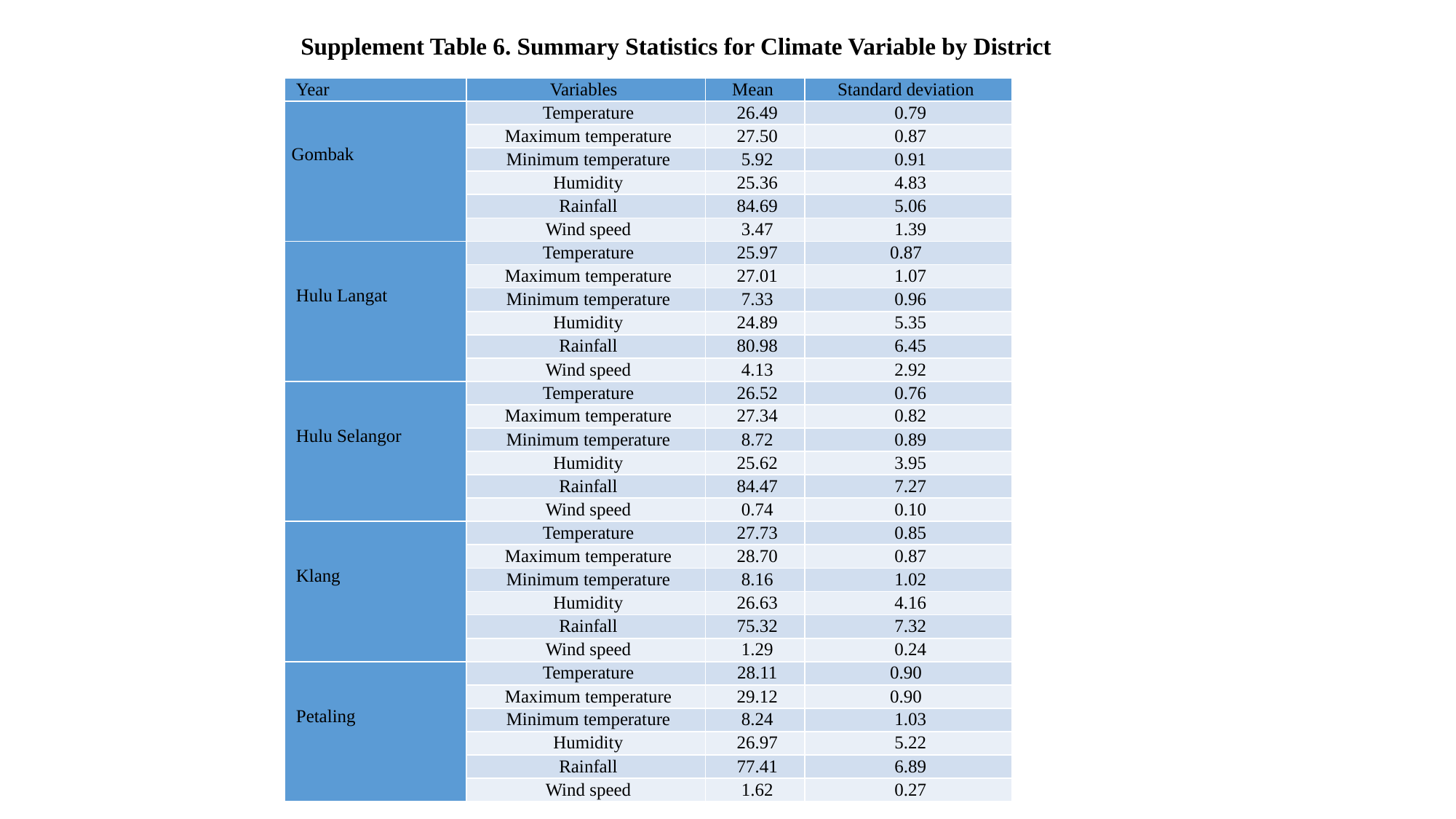

Supplement Table 6. Summary Statistics for Climate Variable by District
| Year | Variables | Mean | Standard deviation |
| --- | --- | --- | --- |
| Gombak | Temperature | 26.49 | 0.79 |
| | Maximum temperature | 27.50 | 0.87 |
| | Minimum temperature | 5.92 | 0.91 |
| | Humidity | 25.36 | 4.83 |
| | Rainfall | 84.69 | 5.06 |
| | Wind speed | 3.47 | 1.39 |
| Hulu Langat | Temperature | 25.97 | 0.87 |
| | Maximum temperature | 27.01 | 1.07 |
| | Minimum temperature | 7.33 | 0.96 |
| | Humidity | 24.89 | 5.35 |
| | Rainfall | 80.98 | 6.45 |
| | Wind speed | 4.13 | 2.92 |
| Hulu Selangor | Temperature | 26.52 | 0.76 |
| | Maximum temperature | 27.34 | 0.82 |
| | Minimum temperature | 8.72 | 0.89 |
| | Humidity | 25.62 | 3.95 |
| | Rainfall | 84.47 | 7.27 |
| | Wind speed | 0.74 | 0.10 |
| Klang | Temperature | 27.73 | 0.85 |
| | Maximum temperature | 28.70 | 0.87 |
| | Minimum temperature | 8.16 | 1.02 |
| | Humidity | 26.63 | 4.16 |
| | Rainfall | 75.32 | 7.32 |
| | Wind speed | 1.29 | 0.24 |
| Petaling | Temperature | 28.11 | 0.90 |
| | Maximum temperature | 29.12 | 0.90 |
| | Minimum temperature | 8.24 | 1.03 |
| | Humidity | 26.97 | 5.22 |
| | Rainfall | 77.41 | 6.89 |
| | Wind speed | 1.62 | 0.27 |

## Slide 7
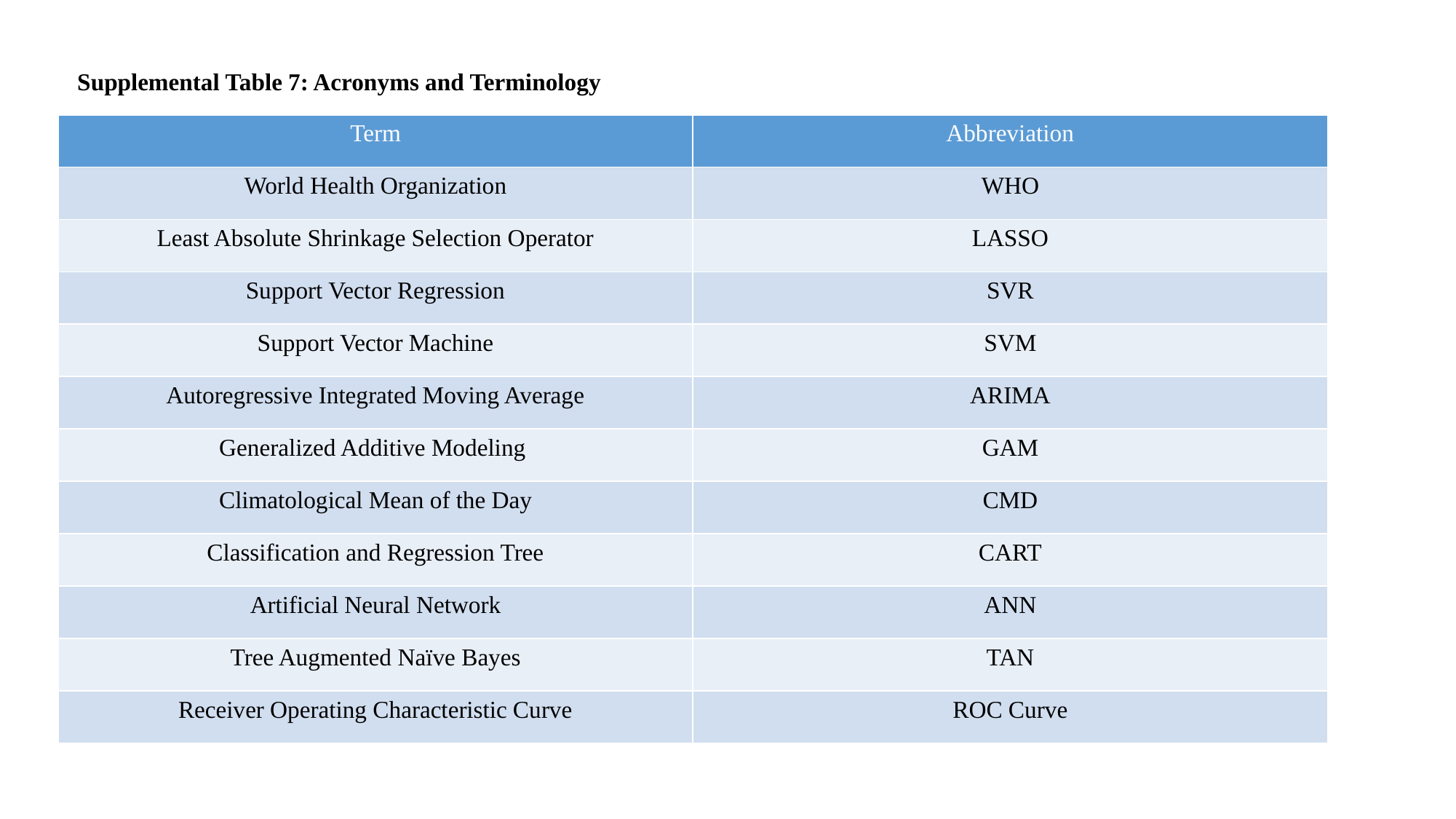

Supplemental Table 7: Acronyms and Terminology
| Term | Abbreviation |
| --- | --- |
| World Health Organization | WHO |
| Least Absolute Shrinkage Selection Operator | LASSO |
| Support Vector Regression | SVR |
| Support Vector Machine | SVM |
| Autoregressive Integrated Moving Average | ARIMA |
| Generalized Additive Modeling | GAM |
| Climatological Mean of the Day | CMD |
| Classification and Regression Tree | CART |
| Artificial Neural Network | ANN |
| Tree Augmented Naïve Bayes | TAN |
| Receiver Operating Characteristic Curve | ROC Curve |
